# A one-pot organocatalytic process for the synthesis of cyclic carbonates from CO_2_ and alkenes using cumene hydroperoxide as a green oxidant

**DOI:** 10.1039/d5gc06899a

**Published:** 2026-03-03

**Authors:** Angelo Scopano, Nicole Potenza, Giovanni Berluti, Remco W. A. Havenith, Arjan W. Kleij, Paolo P. Pescarmona

**Affiliations:** a Engineering and Technology Institute Groningen, University of Groningen (UG) Nijenborgh 3 9747 AG Groningen The Netherlands p.p.pescarmona@rug.nl; b Institute of Chemical Research of Catalonia (ICIQ-Cerca) Avenida Països Catalans 16 43007 Tarragona Spain; c Stratingh Institute for Chemistry and Zernike Institute for Advanced Materials, University of Groningen (UG) Nijenborgh 3 9747 AG Groningen The Netherlands; d Polykey Polymers, Gipuzkoa science and technology park Miramon Pasealekua 170 20014 Donostia-San Sebastian Spain; e Ghent Quantum Chemistry Group, Department of Chemistry, Ghent University Krijgslaan 281 (S3) B-9000 Gent Belgium; f Catalan Institute of Research and Advanced Studies (ICREA), Pg. Lluis Companys 23 08010 Barcelona Spain

## Abstract

Cyclic carbonates are usually obtained from coupling of carbon dioxide and epoxides. The latter are generally prepared through the selective oxidation of alkenes or other compounds containing a double bond. However, a one-pot route in which an alkene is directly converted into a cyclic carbonate would be preferable as it would circumvent the handling of generally toxic epoxides and would increase process efficiency in terms of energy, solvent and reagents usage. Here, we present an attractive strategy combining a recyclable oxidant (cumene hydroperoxide, CHP) with an inexpensive, metal-free organic halide salt as catalyst. These components act cooperatively promoting the oxidation of the chosen model substrate (styrene) and the cycloaddition of CO_2_ to the generated epoxide intermediate. Tetrabutylammonium bromide exhibited the best catalytic performance, providing a 55% styrene carbonate yield after 6 h at 10 barg of CO_2_ and 80 °C using 1.5 equivalents of oxidant; and 67% in the presence of 4 equivalents of oxidant. These cyclic carbonate yields are significantly higher than those obtained with other oxidants (*tert*-butyl hydroperoxide and hydrogen peroxide). A scope of substrates was converted into their respective cyclic carbonates including a new bio-based methylisoeugenol-derived product and a cyclic carbonate attained from bio-based methyl oleate (having a disubstituted double bond). From mechanistic control experiments, we determined that the oxidation step proceeds through a radical mechanism, with an active involvement of CHP in epoxide activation *via* hydrogen-bonding, demonstrating a dual role of the oxidant. Our strategy offers a practical proof of concept of a direct approach to cyclic carbonates with a simple organocatalyst that could be reused in four consecutive runs with a similar performance, and using a recyclable oxidant.

## Abstract 2

The one-pot synthesis of cyclic carbonates from carbon dioxide and styrenes using a potentially recyclable oxidant was realised with a single, homogeneous quaternary ammonium halide catalyst.

## Graphical abstract

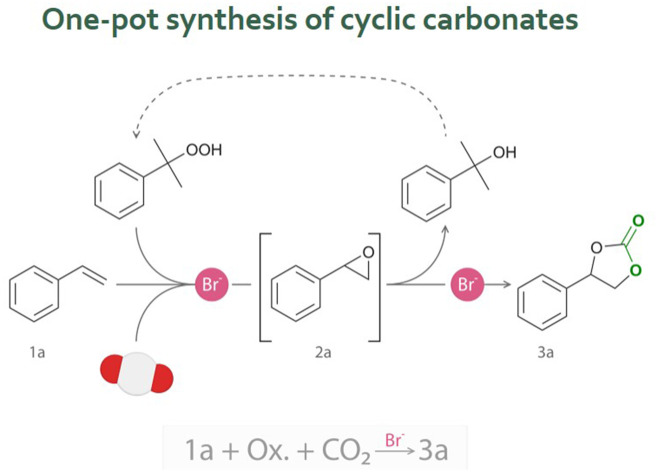

**Green foundation**

1. One-pot synthesis of cyclic carbonates from alkenes is attractive as it bypasses the need for isolating toxic epoxide intermediates. Our work represents a green advance by employing a recyclable and effective oxidant as cumene hydroperoxide (CHP) and a recyclable, metal-free and inexpensive organocatalyst.

2. We demonstrate that the use of CHP in combination with tetrabutylammonium bromide (TBABr) as catalyst leads to much higher cyclic carbonate yield and oxidant efficiency compared to other commonly-used oxidants in the one-pot synthesis from alkenes and CO_2_. The scope of cyclic carbonates includes bio-based compounds never prepared before with a one-pot approach. The side product of CHP and the TBABr catalyst could be recovered and the latter was also effectively recycled.

3. A greener process may be achieved by exploring heterogeneous catalysts in flow reactors. Future work should also quantitatively assess the advantages of our one-pot approach through Life Cycle Assessment.

## Introduction

Cyclic carbonates (CCs) are widely applicable as green solvents, as components of electrolytes and as monomers for polymer synthesis.^1^ They can be obtained through the catalytic cycloaddition of CO_2_ to epoxides with 100% theoretical atom economy.^2^ This transformation valorises carbon dioxide as a renewable carbon feedstock and contributes to the production of added-value chemicals through a circular carbon approach. Cyclic carbonates are conventionally synthesised through a sequence that involves the selective epoxidation of alkenes,††In this work, we will use the term alkenes in a broad sense to refer to compounds containing a double bond, including styrene (phenylethene) and related compounds, and unsaturated fatty acid esters. followed by the cycloaddition of CO_2_ to the epoxide ring.^1,2^ The epoxidation step requires an oxidant (*e.g.* O_2_, H_2_O_2_ or organic peroxides) and, generally, a catalyst, which typically activates the oxidant towards reaction with the double bond.^3^ The epoxidation can also be achieved without a catalyst using a stoichiometric oxidant such as *meta*-chloro-perbenzoic acid (*m*-CPBA). However, this option is less preferable in the context of green chemistry as the peroxy acid is hazardous, non-recyclable, expensive and requires the use of harmful solvents such as dichloromethane or toluene.^4^ Typically, the cycloaddition step proceeds through the ring-opening of the epoxide in the presence of nucleophilic catalysts, and subsequent insertion of carbon dioxide.^5,6^ Several classes of catalysts have been developed for this reaction, including homogeneous and heterogeneous ones. Many of these catalysts combine halide species acting as nucleophiles with Lewis acid sites, either as metal active sites or as metal-free hydrogen bond donors.^1,2,5–7^ Besides the specific challenges for each of the two reactions, which are often addressed by the development of effective catalysts, this approach involves isolation and handling of epoxides, which are generally toxic compounds.^8^

**Fig. 1:**
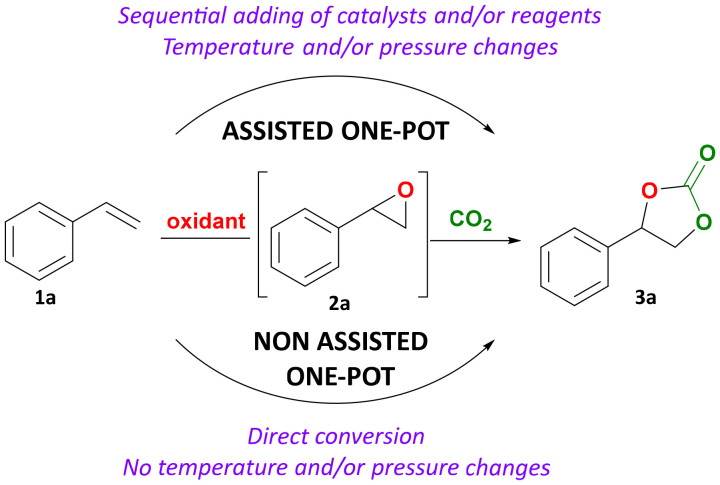
Schematic representation of assisted (top) and non-assisted (bottom) one-pot direct oxidative carbonation of styrene into styrene carbonate, without isolation of the intermediate epoxide.

In this context, it would be attractive to develop one-pot approaches to prepare cyclic carbonates directly from alkenes, thus circumventing the isolation of and exposure to the epoxide intermediates.^1^ In the last two decades, several approaches for the one-pot carbonation of compounds containing a double bond have been reported.^9–13^ Two main approaches can be differentiated (Fig. 1). In the first one (often referred to as a non-assisted protocol), all reactants and catalyst(s) are present in the reactor at the start of the process and the reaction conditions (*e.g.* temperature) are not modified during the process. In the second approach (assisted protocol), the overall process also occurs in one-pot and does not require separation of the epoxide, but it can involve intermediate addition of a catalyst and/or the adjustment of parameters such as temperature and pressure of CO_2_. The latter option is typically chosen when the catalysts and/or reaction conditions for the epoxidation and carbonation steps are not compatible, and in such case the two reactions are typically carried out sequentially.

Green oxidants such as hydrogen peroxide and oxygen would be the preferred choice in one-pot protocols for the direct carbonation of compounds containing double bonds. Their classification as green oxidants arises from their low toxicity and the fact that they generate benign by-products. However, the use of these oxidants generally requires the presence of two distinct catalysts that typically cannot operate simultaneously (*e.g.* halides promote H_2_O_2_ decomposition), which means that the process needs to be conducted under an assisted regime in which the carbonation catalyst is added after the epoxidation step. This feature limits the applicability of these approaches. Additionally, such processes generally do not provide high yields of the cyclic carbonate products.^10^ Moreover, the presence of water cannot be avoided when hydrogen peroxide is used as the oxidant (as H_2_O_2_ is mostly available in aqueous solution and its utilisation as oxidant generates H_2_O as side product), and this may lead to undesired hydrolysis of the epoxide giving a diol by-product.^14,15^

The first reports of one-pot procedures for the synthesis of cyclic carbonates using O_2_ as the oxidant relied on a single metal catalyst (*e.g.* a homogeneous Rh-complex, or niobium oxide, Nb_2_O_3_) but were limited to styrene as substrate with only low yields (5–20%) of carbonate product.^16,17^ More recently, a catalyst based on an imidazolium bromide-functionalised Mn(iii)-porphyrin metal–organic framework was reported to be effective with molecular oxygen as oxidant in a non-assisted protocol to give styrene carbonate with excellent selectivity (95%). However, this process requires the use of an excess of isobutyraldehyde (2 equiv.) as a sacrificial reductant.^18^ The utilisation of H_2_O_2_ is often reported in combination with polyoxometalates or organic halides as catalysts in assisted protocols.^14,15,19–21^ Few examples in this context reported a non-assisted approach, though without addressing the low compatibility between tetrabutylammonium halides and H_2_O_2_.^22–24^

Given the above-described limitations of one-pot processes using H_2_O_2_ as the oxidant, other peroxides having a better compatibility with halides could create new incentives for non-assisted conversion of alkenes into their cyclic carbonates, thus contributing to a more sustainable and scalable practice. In this regard, a common oxidant is *tert*-butyl hydroperoxide (TBHP).^25–27^ This oxidant is attractive in terms of cost and handling when compared to *m*-CPBA, though it is less sustainable and more expensive than H_2_O_2_. Furthermore, the utilisation of TBHP has similar issues as H_2_O_2_ with respect to the presence of water, as it is mainly supplied as an aqueous solution. Additionally, the use of TBHP generates *tert*-butanol as a side-product, which is difficult to separate from the reaction mixture and cannot be easily recycled.^28^

**Fig. 2:**
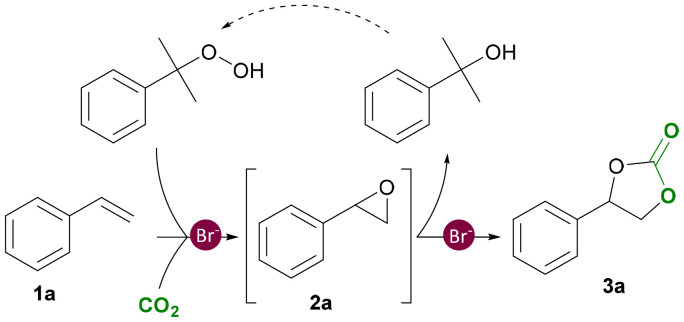
One-pot carbonation through dual bromide catalysis. In the example shown, styrene is converted into styrene carbonate using CO_2_, cumene hydroperoxide (CHP) as oxidant and an organic bromide salt as a metal-free catalyst.

In this work, we report for the first time a more sustainable one-pot strategy for the conversion of styrenes combining CHP as the oxidant and an organic halide as the catalyst under non-assisted and mild reaction conditions (Fig. 2). CHP has several important advantages compared to TBHP: (i) it is considered safer for application at an industrial scale as it has a higher decomposition temperature;^29^ (ii) it is cheaper compared to *m*-CPBA and TBHP;^30,31^ and (iii) its regeneration from the side product 2-phenyl-2-propanol (2P2P, Fig. 2) has been patented and is currently applied at an industrial level in the Sumitomo process.^32^ A wide catalyst screening and a systematic optimisation of the reaction conditions was carried out to maximise the carbonate yield for a broad scope of substrates. This includes the first report of the one-pot conversion of the lignin-derived methyl isoeugenol to its cyclic carbonate.^33^ The catalyst could be recovered and successfully reused in consecutive runs, and pure 2P2P was isolated by column chromatography.

## Experimental section

### Materials

Styrene 1a (≥99% purity, ReagentPlus®), 2-methylstyrene 1b (>96% purity, TCI Europe), 3-methylstyrene 1c (>96% purity, TCI Europe), 4-methylstyrene 1d (>96% purity, TCI Europe), 2-chlorostryene 1e (>96% purity, TCI Europe), 4-chlorostyrene 1f (>98% purity, TCI Europe), α-methyl styrene 1g (>99%, Sigma-Aldrich), methyl isoeugenol 1h (≥98% purity, Sigma-Aldrich), *trans*-stilbene 1i (96% purity, Sigma-Aldrich), methyl-oleate 1j (99% purity, Sigma-Aldrich), mesitylene (>98% purity, Sigma-Aldrich), deuterated chloroform (>99.6% atom %, Sigma-Aldrich) and tetrahydrofuran (THF, ≥99%, HPLC grade, containing inhibitors) were stored according to their PSDSs and used without further purification. Compounds 1a–g contained 4-*tert*-butylcatechol as stabiliser to prevent radical polymerisation. Aqueous hydrogen peroxide 50 wt% (Sigma-Aldrich), cumene hydroperoxide in cumene 80 wt% (Sigma-Aldrich), TBHP 5.0–6.0 M in decane (Sigma-Aldrich) and aqueous TBHP 70 wt% (Sigma-Aldrich) were purchased from Merck and stored at 2–8 °C in a PTFE box and used without further purification. Additionally, TBHP in decane was stored in the presence of molecular sieves (3 Å). All the oxidant reagents contained radical inhibitors. Hexadecyltrimethylammonium bromide (CTABr, ≥98% purity, Sigma-Aldrich), tetrabutylammonium bromide (TBABr, ≥98% purity, Sigma-Aldrich), tetraethylammonium bromide (TEABr, ≥98% purity, Sigma-Aldrich), tetramethylammonium bromide (TMABr, ≥98% purity, Sigma-Aldrich), tetrabutylammonium chloride (TBACl, ≥98% purity, Sigma-Aldrich), aqueous tetrabutylammonium hydroxide 40 wt% (TBAOH, Sigma-Aldrich), 4-butyl-1-methylimidazolium chloride (bmimCl, >99% purity, IoLiTec), 4-butyl-1-methylimidazolium bromide (bmimBr, >99% purity, IoLiTec), and bis(triphenylphosphoranylidene) ammonium chloride (PPNCl, 97% purity, Sigma-Aldrich) were used as catalysts without further treatment. Liquid carbon dioxide, grade 5.0 (99.999% purity) was purchased from Westfalen Gassen Nederland BV and used without any treatment. Milli-Q water was obtained through a Purelab flex Elga series purificator.

### Synthesis of PPNBr

This organic bromide was obtained through ion-exchange following a previously reported procedure starting from the corresponding chloride salt.^34^ Firstly, PPNCl (3.03 g, 5 mmol) was dissolved in 30 mL of milliQ water at 65 °C in a 250 mL round-bottom flask. Subsequently, KBr (10.62 g, 90 mmol) was added to the same solution. The mixture was magnetically stirred at 400 rpm for 45 min. The vessel was then removed from the oil bath and placed into an ice bath for 1 h. The white solid that appeared was recovered by paper filtration and dissolved in 30 mL of acetonitrile. Water was removed from the obtained solution by drying with MgSO_4_. Next, the solid MgSO_4_ was removed by filtration, and the solvent was evaporated from the filtrate in a rotary evaporator. The obtained white solid was rinsed three times with 20 mL of an acetone : diethyl ether mixture (1 : 1 v/v). Finally, the product was dried under reduced pressure in a vacuum oven at 70 °C for 48 h.

### Procedure for the one-pot synthesis

The one-pot tests were performed in a high-throughput experimentation set-up manufactured by Integrated Lab Solutions (ILS) and consisting of: (i) a reactor block allowing to perform 10 reactions simultaneously in individually-stirred batch reactors, and (ii) a stirred batch reactor with a window allowing to observe the phase behaviour. A detailed description of the set-up is available in a previous report.^34^ In a typical test, a chosen amount of catalyst (usually 1 mmol) was weighed using an analytical scale into a 48 mL glass vial containing a 2.0 cm × 0.6 cm magnetic stirring bar. Subsequently, 10 mmol of the substrate and a chosen amount of the oxidant (typically 2.86 g of a cumene hydroperoxide solution, corresponding to 15 mmol of oxidant) was added.

Immediately after the weighing procedure, the glass vial was closed with a rubber cap to avoid any evaporation and the mixture was kept statically for 15 min. This waiting time was maintained as a safety precaution to check potential pressure build up due to oxidant decomposition in the presence of the catalyst. Next, the rubber cap was removed and each glass vial was closed with a screw cap equipped with a silicone/PFTE septum and pierced with two needles.^34^ Then, each vial was placed into the reactor block. The reactor was closed, purged three times with 5 barg of N_2_ and one time with 10 barg of CO_2_ at room temperature, after which the reactor was allowed to depressurise to a CO_2_ pressure between 1 and 2 bar (*i.e.* between 0 and 1 barg). For the reference tests under N_2_ atmosphere, the CO_2_ purging step was skipped. The reactor block was then heated to the desired reaction temperature (typically 80 °C). When the desired temperature was reached, the reactor was pressurised further with CO_2_ (or N_2_) up to the desired pressure. It must be highlighted that the pressures in this work are reported as Gauge pressures (*i.e.* in barg). Next, the stirring (600 rpm) was turned on through the software, and this moment was considered as the beginning of the reaction. At the end of each experiment, the stirring was stopped, the reactor was cooled down and then depressurised. The automatic depressurisation function was applied until the pressure inside the reactor was <10 barg, after which the depressurisation was manually controlled. The reactor block was opened, the vials were recovered, and the needles were removed from the septum cap. Then, mesitylene (0.180 g, 1.50 mmol; ^1^H-NMR internal standard, IS) was added to each reaction vial, after which the content was stirred at 400 rpm for 2 min using a magnetic stirrer, followed by analysis (see next section).

### ^1^H-NMR analysis

^1^H-NMR analysis was used for quantification of reactants and products, using 1.5 mmol of mesitylene as internal standard. This quantity of mesitylene was soluble in most of the reaction crudes. In a few cases, however, THF was gradually added to ensure formation of a single phase. The choice of adding the internal standard after removing the vials from the reactor block and not before the catalytic test was done to avoid any potential oxidation of the IS. The NMR samples were prepared by taking one drop of the reaction mixture (*ca.* 500 µg) and diluting it with 0.40 mL of CDCl_3_ into an NMR tube. The NMR analyses were performed on a Bruker Ascend 600 MHz spectrometer using a quantification protocol (8 scans, delay D1 35 s, acquisition time AQ 5 s). Eqn (1)–(6) were used to determine the amount in moles of each species “i” at the end of the reaction (*n*_i,*t*_), the conversion (*X*), yield (*Y*), mole balance (MB), selectivity (*S*) and CHP efficiency (*E*_CHP_), respectively.1
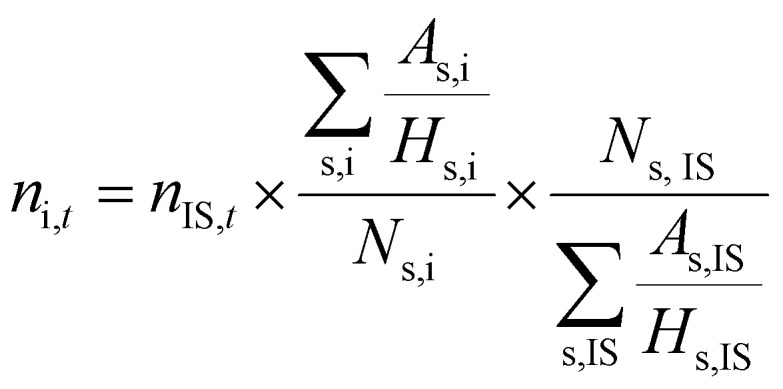
2
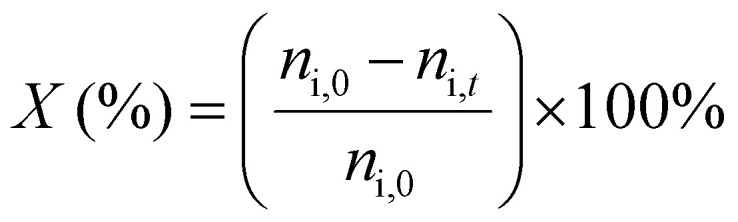
3
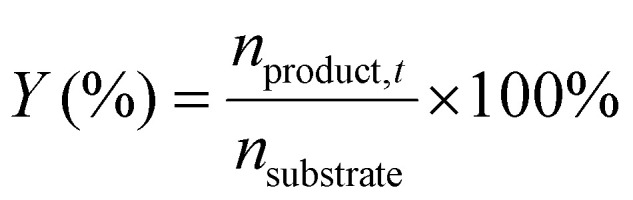
4
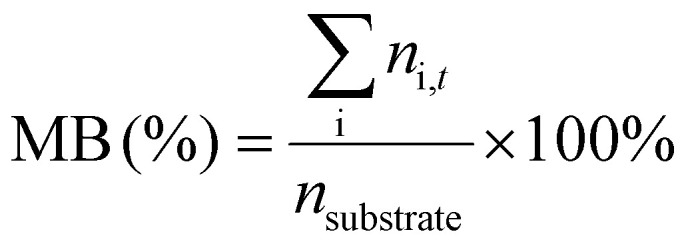
5
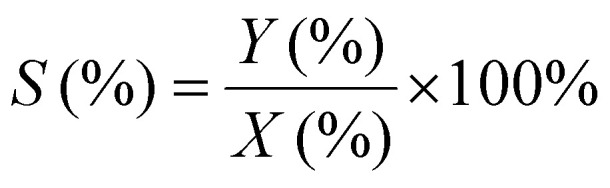
6
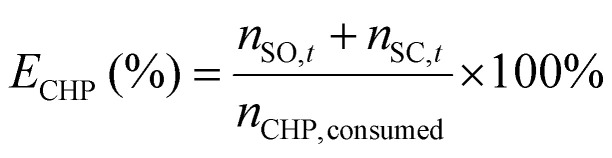
Here, *A*_s,i_ is the area of a signal related to species “i” (for example i = substrate or i = cyclic carbonate product); *H*_s,i_ is the number of protons associated to that signal, *N*_s,i_ is the number of NMR signals related to species “i”; *n*_CHP,consumed_ in eqn (6) was calculated assuming that the only by-products generated from CHP were 2P2P and dicumyl peroxide (DCP).

### GC-MS analysis

GC-MS was used for the identification of the reaction products. About 100 µg of the reaction mixture was placed into a 1 mL glass vial using a glass capillary and diluted with THF (1.5 mL). Each vial was sealed with a crimp cap and analysed with a Hewlett Packard 6890 series GC-MS equipped with an HP-5 column (length: 30 m, internal diameter: 0.32 mm, film thickness: 0.25 µm) and a Hewlett Packard 5973 Mass Selective Detector. Helium was used as carrier gas, with a split ratio of 50. The inlet temperature was 250 °C and the temperature program was from 50 to 300 °C, with a ramp of 10 °C min^−1^.

### Catalyst recycling and 2-phenyl-2-propanol recovery

The reaction mixture at the end of the test with TBABr as the catalyst was transferred into a separation funnel using a minimum amount of dichloromethane to recover any product that remained on the walls of the reaction vial. Subsequently, 2 mL of water per mL of reaction crude (*i.e.* 10 mL for a typical reaction mixture, repeated three times) were added to the separation funnel used to extract the catalyst. At this stage, a colour transition of the organic phase from transparent light yellow to opalescent white could be observed due to the presence of polystyrene. The aqueous and the organic phases were collected in two separated flasks. The aqueous phase was then washed two times with 5 mL of diethyl ether to remove organic residues. Next, the water was evaporated from the resulting transparent solution by means of a rotary evaporator at 40 °C and 50 mbar. These temperature and pressure conditions were reached slowly to avoid violent bubbling of any diethyl ether traces. A light-yellow oil was obtained, which was further dried for 16 h under vacuum (4 mbar) using a Schlenk line. This procedure afforded TBABr as a light-yellow solid with an average yield of 67 ± 15%, which was then used in a subsequent run in which the scale was adjusted to the amount of TBABr. For the recovery of 2P2P, the organic phase previously separated from the aqueous one (containing TBABr) was dried over MgSO_4_, filtered and concentrated. Subsequently, 2-phenyl-2-propanol (*R*_f_ = 0.30) was recovered by mean of a flash silica column chromatography (length: 8 cm, internal diameter: 25 mm) using Silica 60M as stationary phase and dichloromethane as the eluent.

### Density functional theory (DFT) calculations

All DFT calculations were performed using the M06-2X functional combined with the 6-311+G(d,p) basis set using the Gaussian 16 software package.^35^ The thermochemical analysis was performed at temperature of 80 °C and a pressure of 10 bar. The hybrid density functional M06-2X accurately describes thermochemical properties,^36^ while the 6-311 + G(d,p) basis set provides a more complete description including polarisation functions. Given the excess of cumene hydroperoxide utilised in the optimised reaction conditions (1.5 equiv.), its solvation effect was studied using the polarisable continuum model (PCM)^37^ and considering two different dielectric constant values (*ε*_r_ = 5 and 15). Since the dielectric constant value for cumene hydroperoxide is not known, these values were chosen based on the dielectric constant of benzyl alcohol (11.92).^38^ A further refinement of the dielectric constant for this solvent seems unnecessary as the differences between the results with the two *ε*_r_ values are negligible. Frequency analyses were carried out at the same computational level to verify that these optimised structures are genuine minima. Our computational results were compared with the experimental and theoretical data reported in the literature, showing excellent agreement and thus validating the reliability of our approach.^39^

## Results and discussion

### Selection of the oxidant

**Scheme 1:**
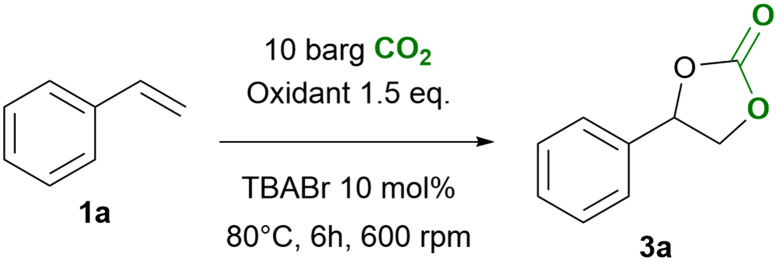
General scheme for the one-pot, direct oxidative carbonation of styrene 1a into styrene carbonate 3a in the presence of an oxidant (CHP, TBHP or H_2_O_2_). Reaction conditions: styrene (10 mmol), oxidant (1.5 equiv.), *p*CO_2_ = 10 barg, TBABr (10 mol%), *T* = 80 °C, *t* = 6 h.

**Fig. 3:**
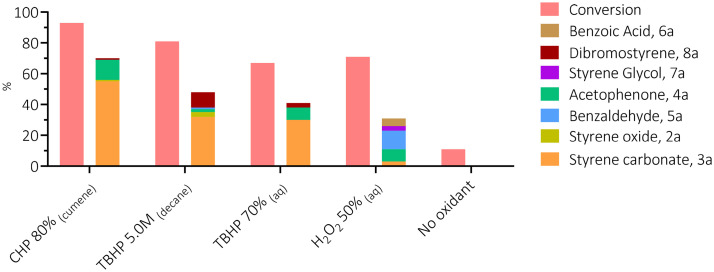
Conversion and product distribution in the oxidant screening for the one-pot conversion of styrene 1a into styrene carbonate 3a. Conversion and selectivity data are reported in Table S2. The incomplete mole balance is caused by the polymerisation of styrene into polystyrene (see the SI, for experimental details, and Fig. S2 and S3).

**Scheme 2:**
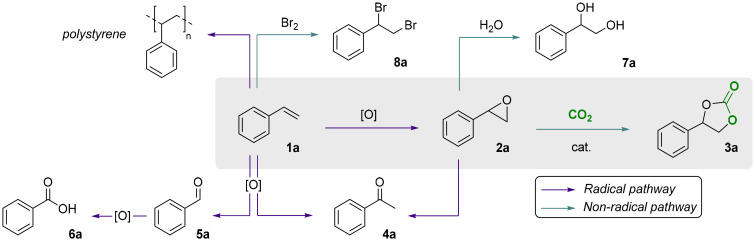
Summary of the desired pathway for the one-pot, direct oxidative carbonation of styrene 1a into styrene carbonate 3a (grey box) and of the main side reaction pathways.

The first step of our investigation consisted in a comparison between different oxidants to investigate their influence on the one-pot, direct carbonation of styrene in the presence of tetrabutylammonium bromide as the catalyst. For this purpose, we selected four oxidants: (i) hydrogen peroxide for its green character, (ii) aqueous *tert*-butyl hydroperoxide (TBHP) and (iii) TBHP in decane, as both are widely-employed organic oxidants in catalytic epoxidation; and (iv) cumene hydroperoxide (CHP) for its industrial applicability and for being recyclable, as demonstrated by the Sumitomo process.^32,40^ We excluded from this screening the peracid *m*-CPBA since it showed violent reactivity when dissolved in styrene in the absence of any organic solvent. The chosen oxidants were used in slight excess (1.5 equiv. relative to styrene) with 10 mol% of tetrabutylammonium bromide as a homogeneous catalyst at 10 barg of CO_2_ at 80 °C for 6 h (Scheme 1). Under these experimental conditions, cumene hydroperoxide was the most effective in the one-pot direct carbonation of styrene to styrene carbonate (SC) 3a, providing a 55% yield of the cyclic carbonate product. This is significantly higher than the yields obtained with TBHP (32% and 30%), and with aqueous H_2_O_2_ (3%), see Fig. 3. The results with TBHP are similar to those previously reported by other groups.^25^ No styrene carbonate was obtained in the absence of an oxidant. Not only CHP showed to be a productive oxidant in terms of styrene conversion and styrene carbonate yield, it also proved to be more chemo-selective (*S*_SC_ = 59%) compared to TBHP (*S*_SC_ = 39–45%). The selectivity for SC 3a in the presence of H_2_O_2_ was the lowest (*S*_SC_ = 4%) in line with the very low styrene carbonate yield. The main cause for this very low selectivity is the formation of oxidation by-products (benzaldehyde, acetophenone and benzoic acid) and, to a lesser extent, the formation of styrene glycol through hydrolysis of styrene oxide (Scheme 2). Styrene is well known to form oxidation products through a radical mechanism.^41^ Additionally, we observed the formation of polystyrene as a side product in all these tests resulting in an incomplete mole balance. The formation of this polymer is supported by the presence of broad peaks in the ^1^H-NMR region for the backbone signals of polystyrene (1.25–2.25 ppm, see the SI, Fig. S2).

Styrene is well known to undergo polymerisation in the presence of peroxides as initiators.^42^ The tendency of polystyrene to form under these conditions was further demonstrated by control experiments, in which the polymer was obtained as a white precipitate using ethanol as anti-solvent (see the SI for further experimental details). The precipitate was shown to be polystyrene by means of ATR-IR (Fig. S1), and displayed similar characteristic ^1^H-NMR signals (Fig. S3) as observed for the crude samples reported in Fig. 3.

Among the oxidants tested in this work, CHP showed the best selectivity for 3a (59% *vs.* 45% for TBHP_(aq)_, Table S2). Although a thorough comparison is hindered by the different reaction conditions, the values of selectivity obtained in this work are comparable to the ones reported in the literature for other one-pot processes occurring in presence of an oxidant and a single catalyst (Table S8). Altogether, the results in Fig. 3 highlight that CHP is a very promising oxidant for the one-pot conversion of styrene to styrene carbonate in the presence of CO_2_, and it was thus selected for further investigation. Aqueous H_2_O_2_, while preferable in terms of greenness, is clearly not a suitable choice under the explored reaction conditions.

### Screening of reaction conditions

The initial screening of the oxidants was carried out with a relatively high catalyst loading (10 mol%). To investigate whether a lower catalyst loading could be adopted and to study the effect of the loading on the reaction performance, we screened different catalyst loadings under the same reaction conditions as used for the oxidant screening (Table 1). In the absence of a catalyst, no styrene carbonate was observed. However, 55% conversion of styrene was observed, together with a 14% yield of styrene oxide and 3% of acetophenone (entry 1). The rest of the conversion was attributed to polystyrene formation.

**Table 1:** Direct oxidative carbonation of styrene 1a into styrene carbonate 3a*via* styrene oxide 2a catalysed by TBABr with different catalyst loadings^a^

Entry	Cat. loading b (mol%)	Conv. 1a b (%)	*Y*_SO_ b (%)	*Y*_SC_ b (%)	Conv. CHP c (%)	*Y*_2P2P_ c (%)	*Y*_DCP_ c (%)	*E*_CHP_ (%)
1	0	55	14	0	16	16	<1	57
2	1	66	25	5	30	24	4	64
3	5	90	9	45	63	48	3	56
4	10	93	1	55	64	52	3	56
5	20	93	0	56	69	55	4	52
6	50	94	0	45	79	49	5	37

a Reaction conditions: styrene (10 mmol), CHP (80% w/w, 15 mmol), TBABr (see table for the loading), *p*CO_2_ = 10 barg, *T* = 80 °C, *t* = 6 h at 600 rpm. Conversion and yields were determined by ^1^H-NMR as described in the experimental section.

b Relative to the initial amount of styrene using mesitylene as an IS.

c Relative to the initial amount of CHP using mesitylene as an IS. The efficiency of CHP towards the formation of styrene oxide and styrene carbonate (*E*_CHP_) was calculated using eqn (6). Abbreviations used: SO stands for styrene oxide (2a), SC for styrene carbonate (3a), CHP for cumene hydroperoxide, 2P2P for 2-phenyl-2-propanol, DCP for dicumyl peroxide, and *Y* represents the yield of the respective product. The results are plotted in Fig. S4a (SI).

In the presence of 1 mol% of catalyst relative to styrene, a slight increase in conversion was observed (66%, entry 2), accompanied by a higher yield of oxidation products (25% of styrene oxide, 5% of acetophenone) and formation of the desired styrene carbonate, though only in low yield and selectivity (*Y*_SC_ = 5%, *S*_SC_ = 8%). These results indicate that the catalyst, as expected, is necessary for the formation of the cyclic carbonate.^43^ The presence of TBABr also promoted the oxidation step, as indicated by the increased yield of styrene oxide (compare entries 1 and 2). This also means an improved selectivity towards the oxidation products compared to the undesired and competitive polystyrene formation. Increasing the catalyst loading to 5 mol% resulted in higher styrene conversion and increased yield and selectivity towards styrene carbonate (entry 3: *Y*_SC_ = 45% and *S*_SC_ = 50%). The lower yield of styrene oxide (9 *vs.* 25%) compared to the test with lower catalyst loading is consistent with the anticipated role of the epoxide as an intermediate in the synthesis of the cyclic carbonate (Scheme 2). This role was confirmed by performing the one-pot catalytic test with a shorter reaction time (2 h), showing that both styrene conversion and styrene carbonate formation increased with the catalyst loading, while the amount of observed styrene oxide decreased (SI, Fig. S4b). When further increasing the catalyst loading (entries 5–6), after 6 h of reaction time nearly no styrene oxide was observed and a maximum yield of styrene carbonate (56%) was reached using 20 mol% of TBABr. An even higher catalyst loading (50 mol%) proved to be detrimental for styrene carbonate yield (entry 6), whereas it resulted in increased yield of acetophenone (from 14% at 20 mol% to 19% at 50 mol% TBABr loading). The observation of lower yields of cylic carbonate and higher yields of acetophenone suggests that catalyst loadings higher than 20 mol% can favour other oxidation pathways. As a result of the screening of the catalyst loading, we selected 10 mol% for the subsequent studies.

Next, we screened the amount of oxidant, as minimising it would enhance the sustainability of the process. In the absence of an oxidant, we observed a non-negligible conversion of styrene (11%), but no oxidation or carbonation products were formed (Table 2, entry 1). Polystyrene was observed as the only product, though its yield could not be accurately quantified by ^1^H-NMR due to line broadening. This observation indicates the tendency of styrene to undergo polymerisation even in the absence of a peroxide initiator. Remarkably, a sub-stoichiometric amount of oxidant (0.5 equiv., entry 2) is sufficient to achieve almost quantitative styrene conversion (96%), though only 16% of styrene carbonate was observed. Acetophenone (6%) and some traces of unidentified impurities were also detected, which together with the remaining styrene (4%) gave a mole balance of only 26%, indicating that 74% of styrene had converted into polystyrene. Further increasing the amount of oxidant (entries 2–6) led to a gradual increase in styrene carbonate yield reaching a maximum value of 67% when 4 equivalents of oxidant were used (entry 6). The increase in the amount of oxidant also led to a decrease in parasitic polystyrene formation as illustrated by increasing MB values, and hence to a higher selectivity towards oxidation products.

**Table 2:** Direct oxidative carbonation of styrene 1a into styrene carbonate 3a catalysed by TBABr in the presence of different amounts of oxidant a

Entry	CHP b (equiv)	Conv. 1a b (%)	*Y*_SO_ b (%)	*Y*_SC_ b (%)	MB b (%)	Conv. CHP c (%)	*Y*_2P2P_ c (%)	*Y*_DCP_ c (%)	*E*_CHP_ (%)
1	0.0	11	0	0	89	—	—	—	—
2	0.5	96	0	16	26	93	49	4	33
3	1.0	89	0	36	58	84	52	3	43
4^d^	1.5	93	1	55	78	64	52	3	56
5	2.0	95	4	63	87	62	50	3	55
6	4.0	100	3	67	87	37	37	3	47

a Reaction conditions: styrene 1a (10 mmol), CHP (80% w/w, 15 mmol), TBABr as indicated, *p*CO_2_ = 10 barg, *T* = 80 °C, *t* = 6 h at 600 rpm. Conversion and yields were determined by ^1^H-NMR as described in the experimental section.

b Relative to the initial amount of styrene.

c Relative to the initial amount of CHP.

d The conditions for entry 4 are equal to those of entry 4 in Table 1. The mole balance (MB) was calculated using eqn (4), by taking into account all the species detected at the end of the reaction derived from styrene but excluding polystyrene, which could not be accurately quantified (see the SI). As such, the mole balance was used as an indicator of the amount of polymer present in the reaction mixture. The efficiency of CHP towards the formation of styrene oxide and styrene carbonate (*E*_CHP_) was calculated using eqn (6).

**Fig. 4:**
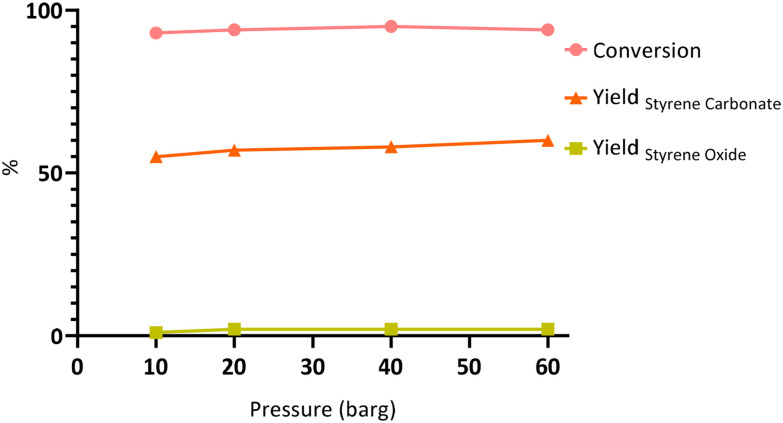
Effect of the CO_2_ pressure on the one-pot oxidative carbonation of styrene 1a into styrene carbonate 3a. Reaction conditions: styrene (10 mmol), CHP (80% w/w, 15 mmol), TBABr (10 mol%), *T* = 80 °C, *t* = 6 h at 600 rpm. The conversion and selectivity data are reported in the SI in Table S3.

We continued our investigation by screening the effect of the CO_2_ pressure on the yield of SC 3a (Fig. 4), as to maximise styrene carbonate selectivity and oxidant efficiency. Increasing the CO_2_ pressure from 10 to 60 barg resulted only in a slight increase of the yield of SC from 55 to 60%, with the selectivity following the same trend (slight increase from 59 to 64%). As the pressure had only a minor effect on the process efficiency, we decided to proceed with 10 barg of CO_2_ pressure.

Prolonging the reaction time up to 18 h led to the expected increase in styrene conversion and styrene carbonate yield (Table 3, entries 1–4). After 18 h, almost quantitative styrene conversion was reached (98%) with a 60% yield of SC 3a. Decreasing the reaction temperature from 80 to 60 °C led to lower styrene conversion and higher amount of (unreacted) styrene oxide intermediate (entry 5). However, when the reaction temperature was increased to 100 °C a different behaviour was observed, with a dramatic increase in the yield of the by-product acetophenone (66%) and only a relatively low yield of styrene carbonate (30%). This chemo-selectivity shift towards acetophenone is attributed to radical species formed through the decomposition of CHP, which has been reported to be significant at temperatures around and above 100 °C.^44^ Finally, we tested the combination of a higher pressure (60 barg CO_2_) and a longer reaction time (entry 7), and a higher pressure in the presence of an excess of the oxidant (4.0 equiv., entry 8). In both cases, no further improvement in the process outcome was apparent compared to the corresponding tests carried out at 10 barg (entry 4 in Table 3 and entry 6 in Table 2).

**Table 3:** Screening of different reaction temperatures and times for the one-pot oxidative carbonation of styrene 1a into styrene carbonate 3a catalysed by TBABr a

Entry	Temp. (°C)	Time (h)	Pressure (barg)	Conversion (%)	*Y*_SO_ (%)	*Y*_SC_ (%)
1	80	2	10	72	9	37
2	80	4	10	90	3	54
3	80	6	10	93	1	55
4	80	18	10	98	0	60
5	60	6	10	47	6	21
6	100	6	10	96	1	30
7	80	18	60	89	0	57
8^b^	80	6	60	99	0	66

a Reaction conditions: styrene (10 mmol), CHP (80% w/w, 1.5 equiv.), TBABr (10 mol%), CO_2_ (see table for the pressure). Conversion and yields were determined by ^1^H-NMR as described in the experimental section.

b 4.0 equiv. of CHP were used.

### Mechanistic studies

Although the role of the halide in the carbonation step is well-documented,^1,2,5,6,16^ there remain various mechanistic aspects of the one-pot process that need to be elucidated. Therefore, we performed a series of systematic control experiments to investigate: (i) the role of the catalyst in the oxidation step (1a → 2a in Scheme 2), and (ii) the role of the oxidant in the carbonation step (2a → 3a in Scheme 2).

We firstly studied how the **2a** (SO) and **3a** (SC) yields are affected by the catalyst loading in the presence or absence of CO_2_ (Table 4), in the latter case with carbon dioxide being replaced by N_2_. Blank experiments without the TBABr catalyst (entry 1) show that cumene hydroperoxide alone is mildly active as an epoxidation agent giving 8% of styrene oxide 2a, both in the presence and absence of carbon dioxide. With increasing amounts of TBABr (1 to 10 mol%), higher yields of styrene oxide 2a were observed under N_2_ atmosphere (entry 2–4), showing that TBABr catalyses the oxidation of styrene by CHP providing SO. When the same reaction was conducted in the presence of CO_2_, styrene carbonate was observed as a major reaction product (especially at higher TBABr loadings), along with styrene oxide, confirming that TBABr is needed to catalyse the carbonation step.

**Table 4:** Control experiments for the one-pot carbonation of styrene 1a in the presence and absence of CO_2_ a

Entry	TBABr	N_2_	CO_2_		
	(mol%)	*Y*_SO_ (%)	*Y*_SO_ (%)	*Y*_SC_ (%)	*Y*_SO_ + *Y*_SC_ (%)
1	0	8	8	0	8
2	1	14	15	1	16
3	5	21	13	16	29
4	10	28	9	37	46

a Reaction conditions: styrene (10 mmol), CHP 80% w/w (1.5 equiv.), TBABr (amount as indicated), CO_2_ or N_2_, *p* = 10 barg, *T* = 80 °C, *t* = 2 h at 600 rpm. The results in the presence of CO_2_ are plotted in Fig. S4b (SI). Additional catalyst loadings were screened at 6 h of reaction time and are reported in Table 1.

**Fig. 5:**
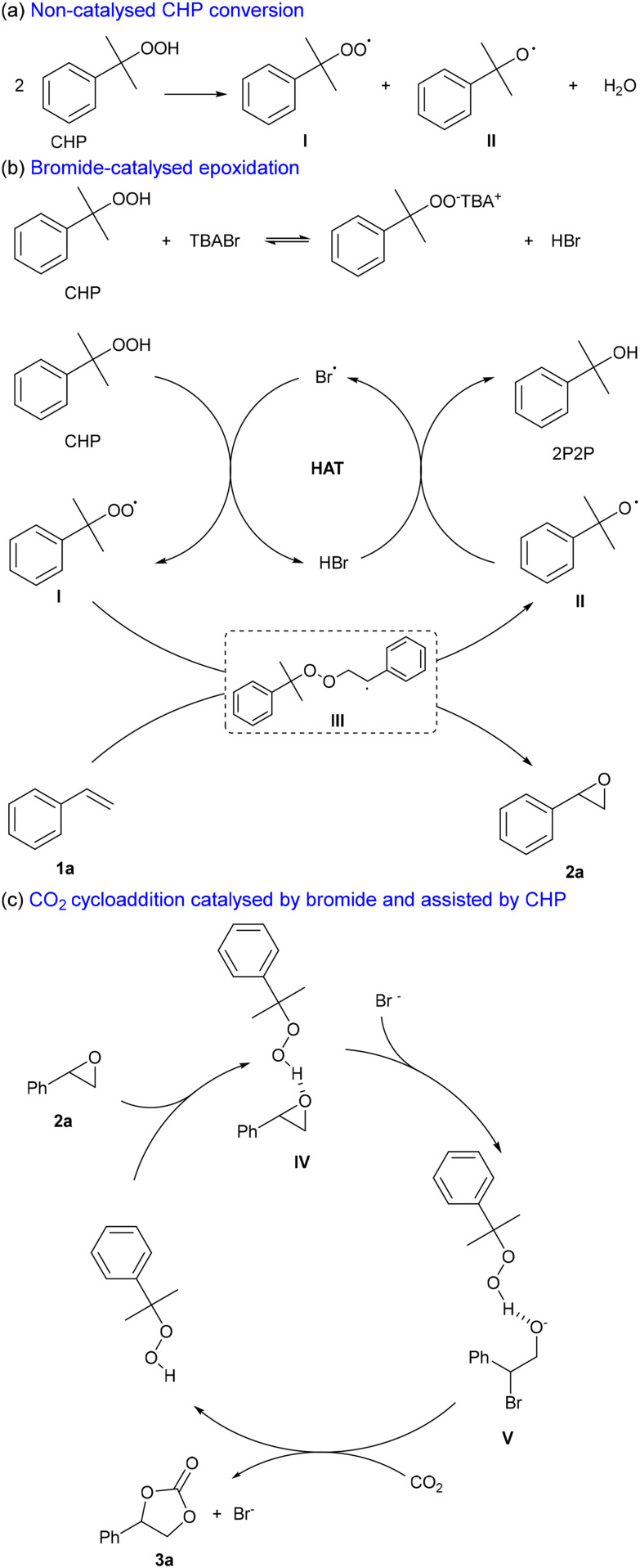
Proposed reaction mechanism for the (a) generation of radical I and II from CHP in absence of any catalyst, (b) bromide-catalysed epoxidation of styrene 1a in the presence of CHP, and (c) carbonation of styrene oxide 2a assisted by CHP acting as a hydrogen bond donor (HBD).

At the same time, the total epoxide/carbonate yield clearly increased with higher catalyst loadings. Upon comparing the yield of SO 2a obtained under N_2_ with the cumulative yields of SO 2a and SC 3a obtained in the presence of CO_2_, higher overall values were observed in the latter case, with the effect being most notable at a TBABr loading of 10% (Table 4). This trend indicates that the presence of CO_2_ has a beneficial effect on the substrate (styrene) conversion. Further insight into the reasons behind this trend will be provided by Density Functional Theory (DFT) calculations (*vide infra*). In addition to the initial control experiments, we performed some inhibition tests (Table 5). The presence of polystyrene observed in the absence of any oxidant, as described above, suggested *in situ* radical formation promoting the polymerisation of styrene (see the SI, Fig. S5 and S6). Therefore, we performed control experiments using two known radical scavengers: TEMPO, as a wide radical scavenger; and IPA as selective hydroxyl radical scavenger.^45^ The addition of TEMPO resulted in significant reaction inhibition, with much lower styrene conversion (Table 5, entry 1) and no observable formation of styrene oxide 2a, indicating that the oxidation step (1a → 2a) proceeds through a radical mechanism. At this stage, it was hypothesised that the mechanism could either involve the peroxy-radical I generated from styrene 1a and CHP, or a hydroxyl radical (OH˙) generated by thermal decomposition of CHP.^44^ To evaluate these two possibilities, we performed an experiment using styrene, CHP and 20 mol% of IPA in the absence of CO_2_ and catalyst (entry 2), and compared the outcome with a blank experiment performed under the same reaction conditions though in the absence of IPA (entry 3). Only a small difference in styrene conversion was observed, indicating that the involvement of hydroxyl radicals in the oxidation is likely negligible. Additionally, we could also exclude that the hydroxyl radical is generated in the presence of the catalyst by carrying out a test with IPA and TBABr in the presence of CO_2_ (entry 4). Comparison between the result of this test with that performed under the same reaction conditions but without IPA inhibitor (see entry 3, Table 3), showed only a small decrease in styrene carbonate yield (49% *vs.* 55%). Based on these two experimental observations, we propose that the reaction proceeds through the formation of the peroxy-radical I derived from CHP, which can occur both in a non-catalytic (Fig. 5a) and a catalytic (Fig. 5b) way. The catalytic mechanism is proposed to proceed with a bromide radical acting as a hydrogen atom transfer (HAT) catalyst (Fig. 5b).^46^ The peroxy-radical I adds to styrene **1a** and generates styrene oxide **2a** and cumyl radical II through intermediate radical III, in agreement with related mechanisms proposed in the literature.^47–49^ Finally, radical II is converted to 2P2P through H-abstraction from HBr leading to the regeneration of the Br˙ radical. Within this scenario, HBr/Br˙ and radicals I and II are continuously regenerated and thus operate in a catalytic regime.

**Table 5:** Radical inhibition tests a

Entry	CO_2_/N_2_	TBABr (mol%)	Additive (mol%)	Conv. (%)	*Y*_SO_ (%)	*Y*_SC_ (%)
1	CO_2_	10	TEMPO, 20	31	0	0
2	N_2_	0	IPA, 20	50	14	0
3	N_2_	0	—	55	16	0
4	CO_2_	10	IPA, 20	90	2	49

a Reaction conditions: styrene (10 mmol), CHP (15 mmol), TBABr (amount as indicated), CO_2_ or N_2_, *p* = 10 barg, *T* = 80 °C, *t* = 6 h at 600 rpm. TEMPO stands for the free radical 2,2,6,6-tetramethyl-1-piperidinyloxy, IPA stands for isopropyl alcohol.

Next, we investigated whether the oxidant can also act as a promoter for the carbonation step (2a → 3a). Four cycloaddition experiments were carried out adding variable amounts of CHP (1, 2.5, 5 and 10 mol% relative to styrene oxide) to a reaction mixture containing styrene oxide (2a) and TBABr (10 mol%). Remarkably, the presence of 1 mol% of CHP results in a notable increase in styrene carbonate yield from 55% without CHP to 80% with CHP (Fig. S7, SI). Increasing the concentration of CHP up to 10 mol% allowed achieving full conversion and selectivity for styrene carbonate 3a, while performing the same experiment in the presence of a much larger amount of CHP (*i.e.* as the one used in our standard one-pot experiments) gave a lower yield of 3a (90%, see Fig. S7) due to competitive (oxidative) side reactions of styrene oxide. These results indicate that CHP significantly contributes to the carbonation reaction, most likely acting as a H-bond donor (HBD) co-catalyst as illustrated in Fig. 5c. It is known that H-bond donors, such as phenol, can promote the carbonation step by interacting with the epoxide, thereby activating the molecule towards ring-opening.^34^

To gain further insight into the observed influence of CO_2_ on the cumulative yields of SO 2a and SC 3a (*vide supra*), we performed Density Functional Theory (DFT) calculations to investigate the thermodynamics (*i.e.* Δ_r_*G*, Δ_r_*H* and Δ_r_*S*) of the overall reaction (1a → 3a) and of each of the individual steps (oxidation, 1a → 2a, and carbonation, 2a → 3a) under 10 bar of CO_2_ and at 80 °C (see Table 6 for details). The computational analysis shows that the initial step, in which cumene hydroperoxide oxidises the double bond of styrene leading quantitatively to styrene oxide (1a → 2a), is thermodynamically very favourable with a Δ_r_*G* of −48.22 kcal mol^−1^ (Table 6, entry 1), and consequently a very large equilibrium constant (*K*_eq_ = 7.2 × 10^29^). Whereas the enthalpy gain (Δ_r_*H*) here is large, the entropic contribution (*T*Δ_r_*S*) is small, as the system transitions from three reactants to three products. In the subsequent reaction step (2a →3a), CO_2_ inserts into the styrene oxide to form styrene carbonate with a relatively modest Δ_r_*G* of −4.09 kcal mol^−1^ (entry 2), which results in a *K*_eq_ of 340. The relatively small Δ_r_*G* of this step results from the combined contribution of a favourable enthalpy term (Δ_r_*H* = −16.27 kcal mol^−1^), and an unfavourable entropic term (*T*Δ_r_*S* = −12.19 kcal mol^−1^). This favourable enthalpy is attributed to the formation of the less strained five-membered ring in styrene carbonate compared to the three-membered ring in styrene oxide. The decrease in entropy is related to the conversion of three reaction components into two final products. Finally, we calculated the thermodynamic values for the overall reaction (1a → 3a), obtaining a Δ_r_*G* of −52.31 kcal mol^−1^ due to a highly favourable enthalpy contribution (Δ_r_*H* = −66.09 kcal mol^−1^) and a moderately unfavourable entropic contribution (*T*Δ_r_*S* = −13.79 kcal mol^−1^).

**Table 6:** DFT calculations for the conversion of styrene 1a into styrene carbonate 3a. The atom balance is neutral in each step a

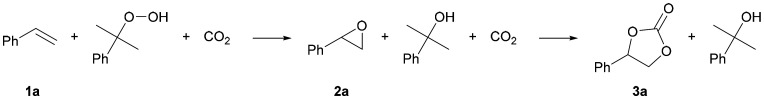							
Entry	Reaction	*ε*_r_ = 5			*ε*_r_ = 15		
		Δ_r_*G*	Δ_r_*H*	*T*Δ_r_*S*	Δ_r_*G*	Δ_r_*H*	*T*Δ_r_*S*
1	1a → 2a	−48.22	−49.82	−1.60	−48.24	−49.96	−1.72
2	2a → 3a	−4.09	−16.27	−12.19	−4.70	−16.88	−12.18
3	1a → 3a	−52.31	−66.09	−13.79	−52.94	−66.84	−13.90

a All calculations were performed in Gaussian 16 using M06-2X/6-311+G(d,p) as described in the experimental section, at *T* = 80 °C and *p*CO_2_ = 10 bar. Δ_r_*G*, Δ_r_*H* and *T*Δ_r_*S* values are reported in kcal mol^−1^, with Δ_r_*G* = *Δ_r_*H**− *T*Δ_r_*S*.

To simulate a potential solvation effect of CHP, as this reactant is present in excess compared to styrene, two different values for the dielectric constant of the medium were used in our computational model. Increasing the dielectric constant from 5 to 15, however, showed only a minimal effect on the thermodynamic parameters with very similar trends. Taken together, the calculations provide evidence that the initial oxidation step (1a → 2a) is thermodynamically very favourable, implying that the equilibrium concentration of SO 2a is not enhanced by its subsequent conversion into SC 3a in the presence of CO_2_. Therefore, we infer that the presence of CO_2_ primarily affects the cumulative yields of SO 2a and SC 3a because it promotes the conversion of SO to SC, thus preventing potential competitive side reactions of SO such as the formation of acetophenone. This hypothesis is supported by literature reports that revealed that styrene oxide can be converted into acetophenone under similar reaction conditions.^48^

### Catalyst screening and substrate scope

**Fig. 6:**
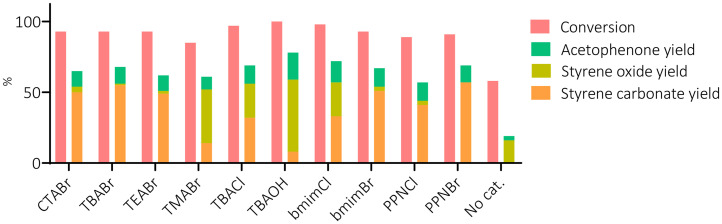
One-pot, direct oxidative carbonation of styrene 1a into styrene carbonate 1c in the presence of different organocatalysts. Reaction conditions: styrene (10 mmol), CHP (15 mmol), *p*CO_2_ = 10 barg, *T* = 80 °C, *t* = 6 h at 600 rpm. The catalysts were used as such in 10 mol% except for tetrabutylammonium hydroxide (TBAOH), which was used as a 40 wt% aqueous solution in 3.75 mol%. Abbreviations used: CTABr = cetyltrimethylammonium bromide, TBABr = tetrabutylammonium bromide, TEABr = tetraethylammonium bromide, TMABr = tetramethylammonium bromide, TBACl = tetrabutylammonium chloride, bmimX = *n*-butyl-methyl-imidazolium halide, PPNX = bis(triphenylphosphine)iminium halide (X = Br, Cl). Conversion and yields were determined by ^1^H-NMR as described in the experimental section.

To expand the catalyst scope, we screened a series of metal-free catalysts in a high-throughput reactor unit that allows reliable comparison (see the SI for details). A series of 10 organic salt catalysts (at 10 mol% loading) was examined to study the effect of both the anion and cation on the one-pot reaction with styrene as a model substrate using 1.5 equiv. of CHP at 10 barg CO_2_, 80 °C during 6 h (see Fig. 6 and the SI, Table S4). The selected organocatalysts are solid at room temperature and completely soluble in the reaction mixture, except for TBAOH which is available as a 40 wt% aqueous solution. As a consequence, the latter forms a biphasic reaction mixture.

We also attempted the use of iodide salts as catalysts, but these compounds led to a violent reaction in the presence of CHP under the optimised reaction conditions. Within 5–10 min after adding the iodide salts to the reaction mixture, a highly exothermic reaction occurred with hot vapour formation. In a closed reaction vessel this could lead to safety issues and thus the combination of iodide and peroxides was avoided. Among the tested catalysts, the conversion of styrene was in a relatively narrow range between 85–100%, while the chemo-selectivity towards styrene carbonate differed significantly. The highest selectivity was obtained in the presence of PPNBr (*S*_SC_ = 63%) and the lowest (*S*_SC_ = 16%) with TMABr (Fig. 6).

We observed that the nature of both the cation and the anion affected the catalytic performance. To rationalise these effects, we compared the catalytic behaviour of catalysts with either a different organic cation or halide. Between the two tested types of halide salts, the bromide-containing ones showed higher selectivity for styrene carbonate compared to the chloride salts (see also the SI, Table S4). These results follow the typical reactivity trend observed for halides in the cycloaddition of CO_2_ to epoxides.^34^ Therefore, we infer that the higher cyclic carbonate selectivity in the presence of bromide-based salts is a result of a higher relative rate of the conversion of styrene oxide (2a) into styrene carbonate (3a) compared to the rate of competitive side reactions. Among the tested cations, PPN^+^ shows the best selectivity towards styrene carbonate (Fig. 6). However, only a 10% difference between the lowest and the highest chemo-selectivity was observed (53% *vs.* 63%) when comparing the relative performance of bromide salts CTABr, TBABr, TEABr, bmimBr and PPNBr, indicating that an anion effect prevails in this two-step one pot formation of styrene carbonate. The only exception among the series of tested bromide salts is TMABr, which shows much poorer carbonate selectivity (*S*_SC_ = 16%) compared to the other quaternary ammonium salts. A plausible cause is the stronger ion pairing between the smaller TMA cation and bromide, thereby lowering the nucleophilic character towards ring-opening of the intermediate styrene oxide.^50^ A similar reactivity trend among the bromide salts was noted when the reaction was monitored at different reaction times (SI, Fig. S8), hence excluding the possibility of catalyst decomposition being responsible for the differences in performance. We also tested aqueous TBAOH as a catalyst under slightly different reaction conditions (Fig. 6). Notably, full conversion but very low selectivity towards styrene carbonate and instead a remarkable selectivity for styrene oxide (51%) was observed. This suggests that a different mechanism is followed with TBAOH. Although interesting, further investigation of the behaviour of TBAOH is outside the scope of this work, which focuses on producing the cyclic carbonate.

**Fig. 7:**
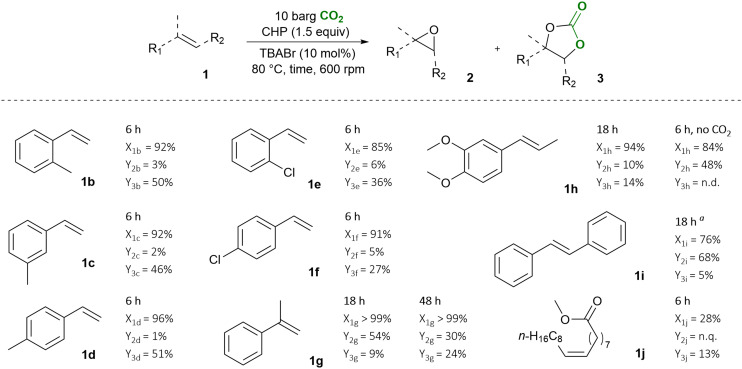
Reaction substrate scope and yields for the corresponding epoxides 2 and cyclic carbonates 3. Reaction conditions: substrate (10 mmol), CHP (15 mmol), TBABr (1 mmol), *p*CO_2_ = 10 barg, *T* = 80 °C at 600 rpm. ^*a*^ THF (3 mL) was used as solvent since *trans*-stilbene is a solid. n.d. stands for not detected, n.q. for not quantified. *X* stands for substrate conversion, *Y* for yield.

Next, we screened different substrates to test the versatility of our catalytic system (Fig. 7). Despite the best performing catalyst being PPNBr, we selected TBABr because its activity is only slightly inferior but it has the advantage of being less expensive and commercially available. This catalyst proved effective in the conversion of various styrenes, and a methyl substitution in *ortho*, *meta* or *para* on the aromatic ring (1b–d) did not significantly affect the cyclic carbonate yield, which ranged from 46% for 3c to 51% for 3d. These yields are the in the same range as observed for SC 3a (55%, entry 4 in Table 1). A chloride substitution on the aromatic ring (1e and 1f) caused a decrease in the cyclic carbonate yield (3e: 27%, 3f: 36%).

We further investigated the effect of a methyl substitution on the vinyl group of styrene (compounds 1g and 1h). If this substitution is in α-position, it is beneficial for the oxidation step leading to full conversion of 1g. However, the increased steric hindrance around the double bond hampers the conversion of α-methyl styrene oxide 2g into 3g. As a consequence of these two effects, only a moderate cyclic carbonate yield was observed (3g: 24% after 48 h), with epoxide 2g being the major product (30%).

The one-pot conversion of bio-based, lignin-derived methyl isoeugenol (1h) to the corresponding cyclic carbonate (3h) was explored here for the first time. This is a challenging substrate as it features methoxy substituents on the aromatic ring and features a less accessible internal double bond. Consequently, a relatively low yield was achieved for methyl isoeugenol carbonate (14% of 3h), accompanied by 10% of the epoxide 2h with 94% substrate conversion after 18 h. Several side products were observed by ^1^H-NMR and GC-MS, though their identification remained elusive (SI, Fig. S9A and B). If the reaction is carried out under the same conditions but in the absence of CO_2_, a relatively good yield of the epoxide 2h can be achieved (48% after 6 h). The combined observations for the conversion of substrate 1h suggest that a methyl substitution on the double bond favours epoxidation, but hinders the successive carbonation. This is in line with the reported lower reactivity of internal epoxides towards CO_2_ cycloaddition,^51,52^ and a higher risk of parasitic side-product formation. Despite the relatively low yield, this is the first reported synthesis of cyclic carbonate 3h from the bio-based methyl isoeugenol.

The substrate scope was expanded to *trans*-stilbene 1i as this compound combines the effect of steric hindrance around the double bond and benzylic radical stabilisation. We observed high epoxide yield (2i: 68%) but virtually no cyclic carbonate was detected (3i: 5%) after 18 h. While the presence of two phenyl substituents does promote epoxidation, it basically hampers the cycloaddition of CO_2_. This follows previous observations with various catalyst systems (including metal-based ones), for which the conversion of *trans*-stilbene oxide to its corresponding carbonate has been rarely productive.^53^

Finally, we investigated whether a compound in which the double bond does not have a phenyl substituent can also be converted with our one-pot approach. We chose methyl oleate 1j as substrate as it is a bio-based compound with a long aliphatic chain and it has a higher boiling point than the reaction temperature. Although only a low cyclic carbonate yield was observed (3j: 13%), the selectivity towards this product was relatively high (46%). In this reaction we were not able to fully quantify epoxide formation (2j) because of signal overlapping in the ^1^H-NMR, but the outcome of this experiment demonstrates that the scope of our one-pot strategy is not limited to styrene and related compounds and could be potentially used for more demanding (aliphatic) alkenes.

### Catalyst recyclability, 2-phenyl-2-propanol recovery and oxidant efficiency

**Fig. 8:**
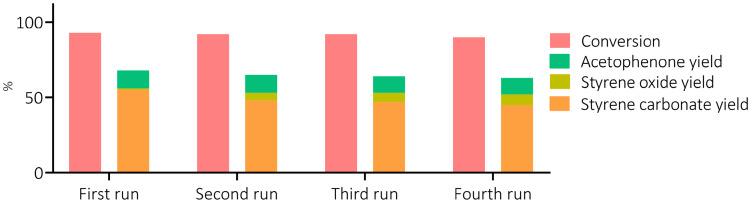
Catalyst recycling studies comprising of four consecutive runs. Reaction conditions: styrene (10 times the molar amount of the recovered catalyst), CHP (1.5 equiv. with respect to styrene), recycled TBABr (10 mol%), *p*CO_2_ = 10 barg, *T* = 80 °C, *t* = 6 h at 600 rpm.

To evaluate the key sustainability aspects of our one-pot process, we set out to recover and recycle the TBABr catalyst by water extraction. On average, a 67 ± 15% mass recovery was achieved after every run. Besides material loss during the separation process, TBABr can also undergo degradation through two possible mechanisms. First, it can undergo intramolecular nucleophilic substitution causing the generation of tributylamine and butyl bromide (SI, Fig. S10), which were both observed by GC-MS. Second, TBABr can suffer from oxidative decomposition.^54,55^ The recovered TBABr catalyst could be reused with only a minor decrease in cyclic carbonate yield and selectivity after the first cycle, and no further loss of catalytic performance in the following cycles (see Fig. 8 and the SI, Table S5).

Besides the recycling of the catalyst, it is also relevant to explore whether it is possible to separate and recover 2P2P from the reaction mixture. This is the product generated from CHP after oxygen-transfer (Fig. 5), and any recovered 2P2P can be reconverted into CHP following a procedure described in a patent and applied industrially.^40^ From a typical reaction mixture (10 mol% of TBABr and an initial amount of 1.5 equiv. of CHP relative to styrene), we were able to recover 86% of the 2P2P formed during the catalytic test, isolating the compound in >90% purity (by ^1^H-NMR) *via* column separation.

Finally, we examined the efficiency of CHP used as the oxidant. CHP can potentially undergo decomposition through different pathways, which compete with its role as oxidant. One decomposition mechanism leads to 2P2P by comproportionation of CHP with the cumene present as an impurity. The generated 2P2P may undergo an elimination reaction leading to α-methyl styrene and water (SI, Fig. S11). In addition, there are several possible side reactions leading to acetophenone/methanol, phenol/acetone (*via* an acid-catalysed process), and dicumyl peroxide (DCP) by radical homocoupling.^56^ To assess the contribution of these possible side reactions, we performed two control experiments by subjecting CHP to our standard reaction conditions either in the presence or absence of TBABr. The conversion of CHP and the yield of 2P2P and DCP were monitored by ^1^H-NMR utilising the chemical shifts of the methyl groups (SI, Fig. S12A–C). No conversion of CHP was detected in the absence of TBABr, whereas in its presence 64% of CHP was converted into 2P2P as the main product (54%), together with acetophenone (7%) and DCP (3%). No phenol or α-methyl styrene were observed under these conditions. The observed formation of acetophenone indicates that the presence of this compound as product in the one-pot tests might stem both from styrene and CHP.

The oxidant efficiency (*E*_CHP_) towards the formation of the epoxide and the cyclic carbonate was determined based on the initial amount of CHP introduced and the cumulative yield of styrene oxide and styrene carbonate (eqn (6)). The CHP efficiency was largely unaffected by the catalyst loading (*E*_CHP_: 56–64%, see entries 1–4, Table 1), though at very high loadings a gradual decrease in the cumulative yield of styrene oxide and styrene carbonate and thus in CHP efficiency was observed (entries 5 and 6 in Table 1). Similarly, in the tests in which the amount of CHP was varied at constant catalyst loading (see Table 2), the lowest CHP efficiency was observed when the catalyst-to-CHP ratio was the highest (entry 2). This is in line with the increased fraction of styrene undergoing polymerisation that we observed in the experiments with lower CHP amounts (Table 2).

It is noteworthy that the oxidant efficiency value for TBHP in water (*i.e.*, *E*_THBP in water_ = 31%) is much lower compared to the value for CHP (Table 1), thus making the latter a preferred oxidant.

Finally, it is worth noting that in all the experiments reported in Tables 1 and 2, the yield of DCP was generally in the same range (3–4%). This suggests that the decomposition of CHP into DCP is occurring to a similar extent independently of the styrene-to-CHP and catalyst-to-CHP ratios.

## Conclusions

In this work, we demonstrate a greener strategy to achieve the one-pot conversion of CO_2_ and alkenes into their cyclic carbonate products. Compared to previous reports, our approach involves a cheap and commercially available organic halide as a homogeneous catalyst and a recyclable oxidant (cumene hydroperoxide, CHP) that allows achieving enhanced performance compared to other oxidants previously reported in non-assisted one-pot protocols involving a single catalyst.

Through a detailed study of the influence of the catalyst nature, reaction pressure/temperature and relative amount of reaction components, we were able to define the most productive process conditions featuring a relative low operating pressure and amount of oxidant. These conditions were applied to a wide set of substrates (styrenes, bio-based and aliphatic compounds), showing the potential of a one-pot carbonation strategy under metal-free conditions using CHP as the oxidant within a complex mechanistic regime, while minimising radical-based substrate polymerisation.^57^ We further have shown that both the side product derived from the oxidant and the optimum catalyst (TBABr) can be recovered from the reaction mixture and, in the latter case, we proved effective recycling. These features represent clear green advances compared to previously reported strategies for the one-pot, direct carbonation of alkenes.

While the current study should be regarded as a significant step forward, the selectivity and yield for the cyclic carbonate target still require improvement. In this respect, adapting the process to continuous flow operation might prove beneficial. This study also provides mechanistic insights that can be helpful to further improve the one-pot process.

In summary, our work represents one of the few examples of non-assisted one-pot protocols in the presence of a single catalyst under solvent-free conditions, and it is the first study that uncovers the green potential of CHP for this type of challenging, one-pot sequential oxidation-carbonation reactions.

## Author contributions

Angelo Scopano: investigation, conceptualisation, data curation, data discussion, writing and editing. Nicole Potenza: DFT calculation and writing. Giovanni Berluti: data discussion, revision. Remco W. A., Havenith: DFT calculation supervision, revision. Arjan W. Kleij: fund-raising and revision. Paolo P. Pescarmona: supervision, methodology, data discussion, fund-raising, writing and editing, revision.

## Conflicts of interest

There are no conflicts to declare.

## Supplementary Material

GC-028-D5GC06899A-s001

## Data Availability

The data supporting this article are available in the supplementary information (SI). Supplementary information is available. See DOI: https://doi.org/10.1039/d5gc06899a. In the case of the raw data, at figshare (https://doi.org/10.6084/m9.figshare.30800813).
