# Supplementary material for: A one-pot organocatalytic process for the synthesis of cyclic carbonates from CO2 and alkenes using cumene hydroperoxide as a green oxidant
Source: Green Chem. 2026 Mar 3;28(19):8116–30. doi: 10.1039/d5gc06899a (PMC12980576; doi:10.1039/d5gc06899a)
Supplement: GC-028-D5GC06899A-s001 [file GC-028-D5GC06899A-s001.pdf]

Supplementary Information (SI) for Green Chemistry.
This journal is © The Royal Society of Chemistry 2026

Supporting information

A one-pot organocatalytic process for the synthesis of cyclic carbonates from CO_2 and alkenes using cumene hydroperoxide as a green oxidant

Angelo Scopano, Nicole Potenza, Giovanni Berluti, Remco W. A. Havenith, Arjan W. Kleij and Paolo P. Pescarmona*

Contents

A one-pot organocatalytic process for the synthesis of cyclic carbonates from CO_2 and alkenes using cumene hydroperoxide as a green oxidant 1
1. Isolation and characterisation of polystyrene from the reaction mixture 2
2. Effect of TBABr concentration 4
3. Polymerisation control test 5
4. Tables and selectivity 6
5. Influence of CHP on the carbonation reaction 8
6. Performance of different tetra-alkyl substituted bromide salts 9
7. ^{1}H-NMR and GC-MS analysis of the crude mixture for the 3 h synthesis 10
8. TBABr and CHP side-reactions 11
9. ^{1}H-NMR-spectra of relevant compounds and reaction mixtures 12
10. Isolation and NMR spectrum of methylisoeugenol carbonate, 3 h 19
11. Reproducibility 22
12. Comparison with state-of-the-art catalytic systems in the literature 23
13. DFT geometries (xyz) 24
14. References 28

1. Isolation and characterisation of polystyrene from the reaction mixture

To confirm the presence of polystyrene in the reaction mixture, a reaction was conducted in the presence of 10 mmol of styrene and 0.5 eq. of CHP as oxidant, and without CO_2 or catalyst. The isolation procedure was also attempted on reaction done in presence of CO_2 and catalyst, though in those conditions no precipitation was observed. We suspect that this is because the length of polymer chains obtained when styrene is consumed in the presence of carbon dioxide and catalyst was not sufficient for its precipitation (the presence of short chains is suggested by the ^{1}H-NMR analysis, see below).

Isolation procedure

To isolate the polymer after having conducted the control experiment (following the procedure explained in the experimental section using 10 mmol of styrene and 0.5 eq. of CHP as oxidant), 20 mL of ethanol were added to the reaction vial as antisolvent and a white precipitate was observed. After having waited 5 min for the decantation, the white precipitate was filtered over a Büchner funnel and then washed with ethanol (20 mL). The filtered precipitate was put back into the vial and dried in a vacuum oven (4 mbar) at 70 °C for 24 h. Next, an FT-IR sample was taken by putting few mg of the obtained solid on an ATR detector. The polymer was obtained in 44% yield (calculated based on the molecular weight of the monomer).

FT-IR characterisation

An IR Shimadzu ® IRTracer-100 equipped with a Specac ATR detector and LabSolutions IR software was used to acquire the spectrum (Happ-Genzel apodisation, number of scans: 64, resolution: 2, 500-4000 cm^{-1}). IR (ATR): 3023, 3918, 1597, 1498, 1440, 1376, 1359, 1150, 1068, 1021, 900, 695, 679, 539 cm^{-1}.

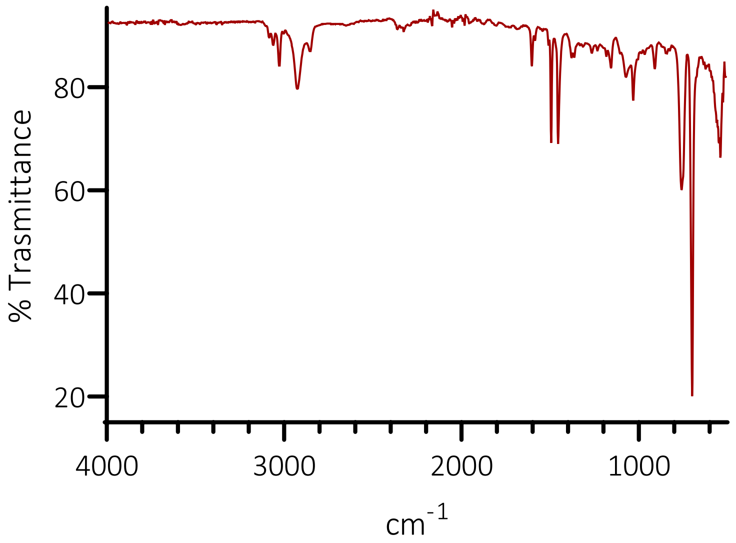

Wave number range	Assignation
3000-3100 cm^{-1}	Aromatic C-H stretches
2800-2950 cm^{-1}	CH_2 asymmetric and symmetric stretches
1450-1600 cm^{-1}	Aromatic ring vibration modes
767 cm^{-1}	Aromatic out of plane ring bend
695 cm^{-1}	Aromatic ring bend

Figure S1. FT-IR spectrum of the isolated polystyrene and corresponding peaks assignment.

^{1}H-NMR spectrum of isolated polystyrene and comparison

The ^{1}H-NMR spectra were acquired using the instrument described in the experimental section. The obtained spectrum matches with the spectra of polystyrene reported in literature.[1] The quantification of the polymer is not feasible through ^{1}H-NMR in our reaction conditions for the following reasons [2,3]: (i) large chain-length dispersion causes NMR peaks overlapping, (ii) the relaxation time of protons located on the polymer depends on its length, and (iii) the peaks of our reaction mixture overlap with the ones of the polymer. **^{1}H NMR (600 MHz, $CDCl_3$)**: 1.41–1.84 (m, 3H), 6.46–7.08 (m, 5H).

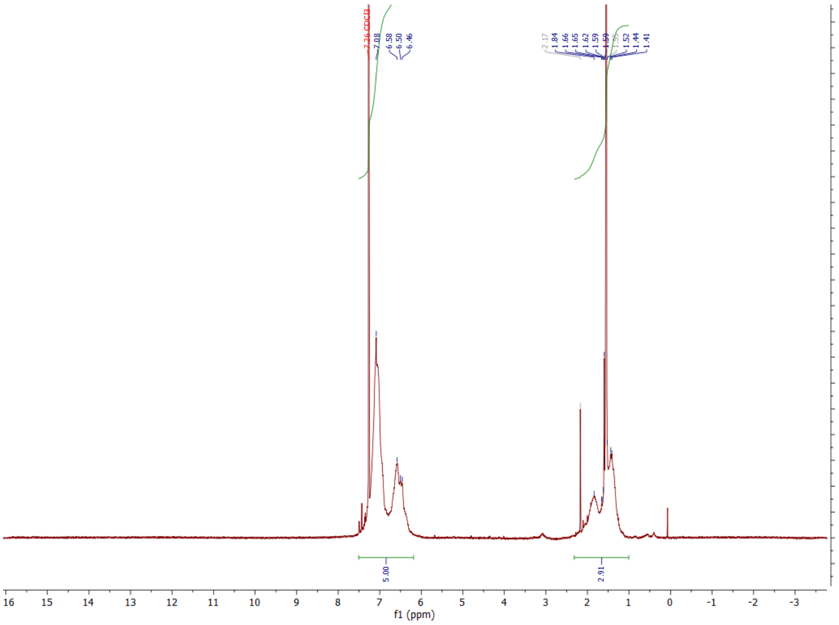

Figure S2. ^{1}H-NMR spectrum of the isolated polymer. $CDCl_3$ was used as deuterated solvent (residual peak at 7.26 ppm). Apart from the peaks of acetone (2.17 ppm) and water (1.56 ppm), the spectrum corresponds to the ones reported in literature for polystyrene. [4,5]

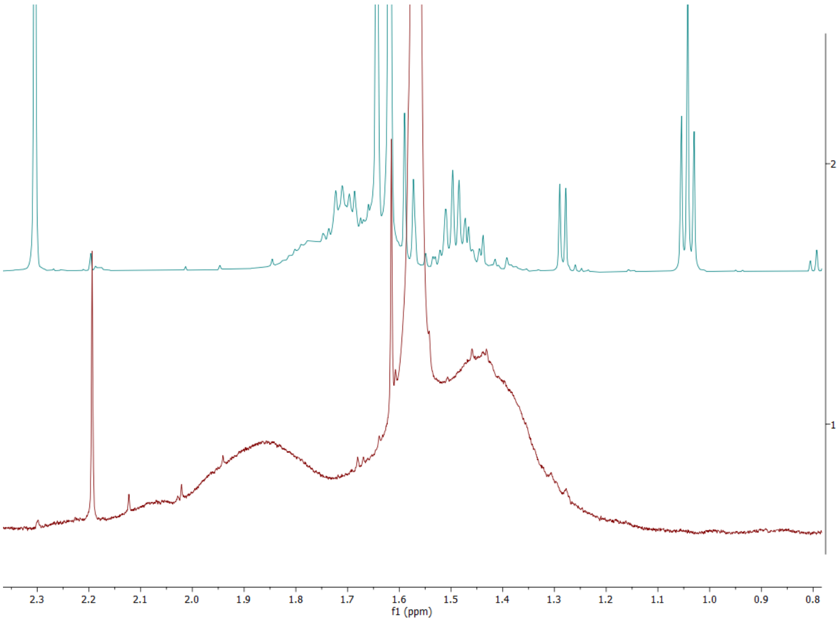

Figure S3. ^{1}H-NMR spectrum of a typical reaction mixture after a test with catalyst and CO_2 (top) and of the isolated polymer after a test without catalyst and CO_2 (bottom). The variations observed for the shift and shape of the peaks are attributed to the average length of the polymer chain which, in the case of the test with catalyst and CO_2, is considerably shorter due to the competitive conversion of styrene to styrene oxide, styrene carbonate and other products.[4]

2. Effect of TBABr concentration

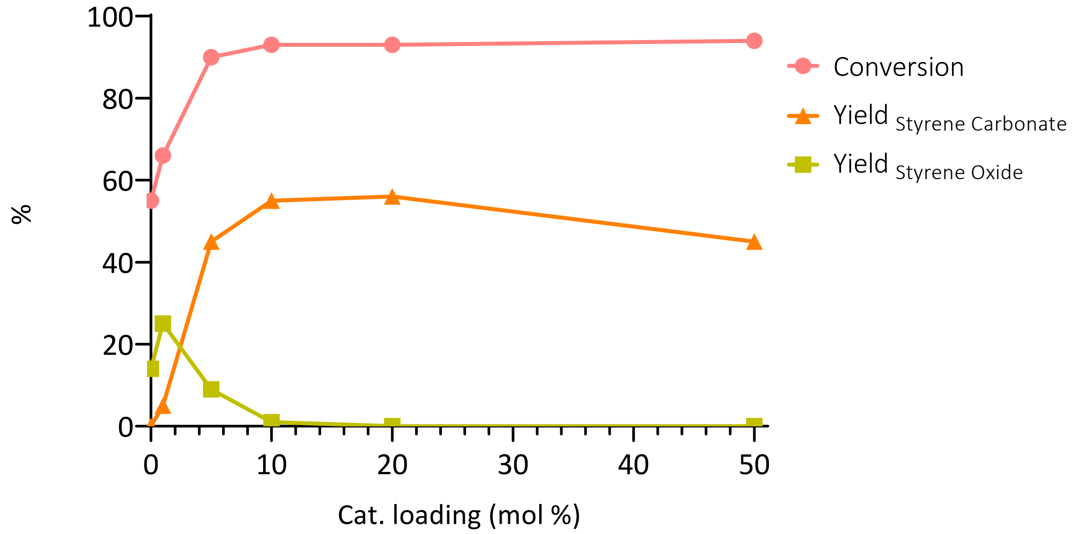

Figure S4a. Effect of TBABr concentration (1-50 mol%) using 6 h as reaction time. The results plotted in this graph are reported in Table 1. Reaction conditions: styrene (10 mmol), CHP (80% w/w, 15 mmol), TBABr as the catalyst (see graph for the loading), T = 80 °C, t = 6 h, 600 rpm.

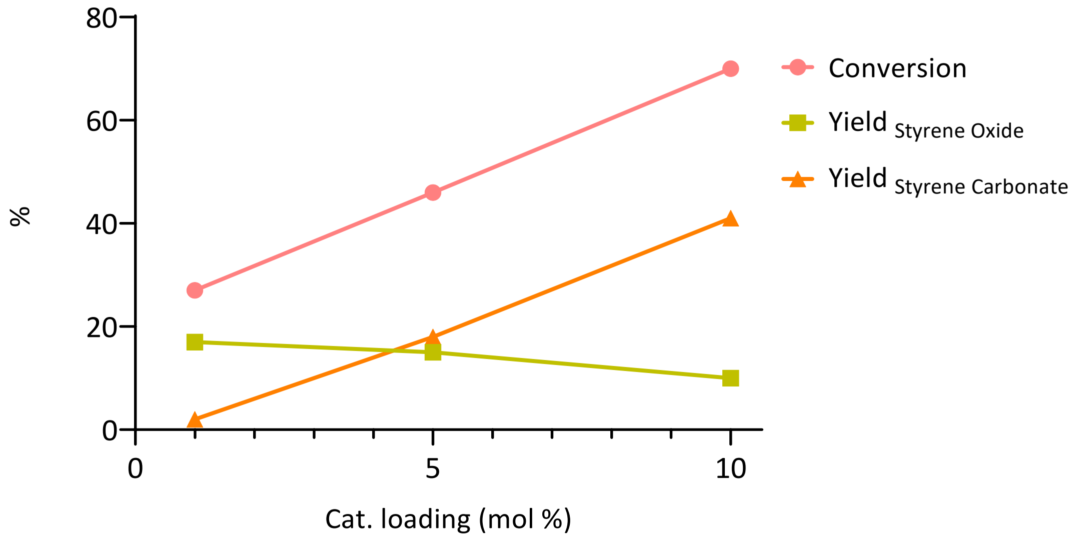

Figure S4b. Effect of the TBABr concentration (1-10 mol%) using 2 h as reaction time. Reaction conditions: styrene (10 mmol), CHP (80% w/w, 15 mmol), TBABr (see graph), T = 80 °C, t = 2 h, 600 rpm. Conversion and selectivity from this graph are reported in Table S6.

3. Polymerisation control test

The result of the control experiment performed in absence of any oxidant and in presence of styrene (10 mmol) and 10 mol% of TBABr is reported in entry 1, Table 2. Styrene and TBABr were mixed at 600 rpm in presence of 10 barg CO_2, at 80 °C for 6 h. As described in the experimental section, even though commercial styrene (i.e. containing polymerisation inhibitors) was used in this experiment, 11% of styrene conversion was observed though none of target products was observed. By analysing the ^{1}H-NMR spectrum of the resulting reaction mixture (Figure S5), a slight broadening of the peaks in the 0.79-2.01 ppm region (similar to the spectrum reported in Figure S2) and an overlapped peak at 1.35 ppm can be observed. The presence of radical initiators derived from cumene hydroperoxide (i.e. $ROO^•$ and $RO^•$) might initiate the radical polymerisation according to the mechanism reported in Figure S6.

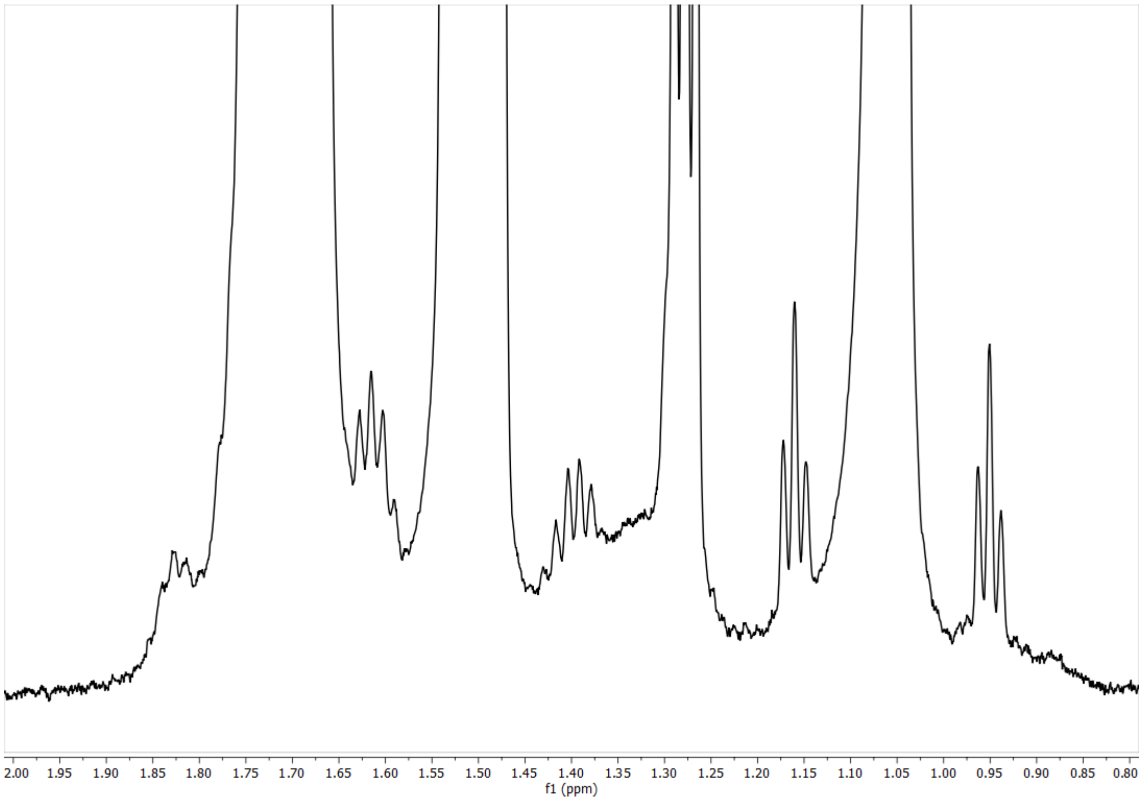

Figure S5. Region 0.79-2.01 ppm of the ^{1}H-NMR (600MHz, $CDCl_3$) spectrum of the crude mixture obtained by mixing styrene and TBABr at 600 rpm, 80 °C for 6 h.

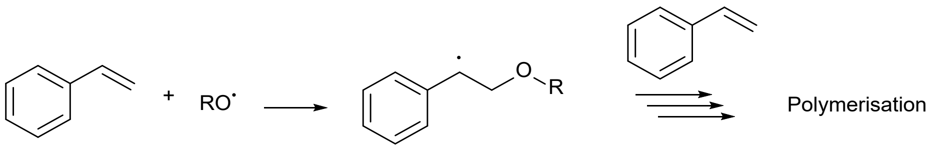

Figure S6. Initiation steps in the radical polymerisation in presence of peroxide as initiator and styrene as monomer.

4. Tables and selectivity

Table S1. Legend from scheme 2.

Code	Substance
**1a**	Styrene
**2a**	Styrene oxide
**3a**	Styrene carbonate
**4a**	Acetophenone
**5a**	Benzaldehyde
**6a**	Benzoic acid
**7a**	Styrene glycol
**8a**	Dibromo styrene

Table S2. Selectivity from Figure 3.

Oxidant	X (%) **1a**	S (%) **2a**	S (%) **3a**	S (%) **4a**	S (%) **5a**	S (%) **6a**	S (%) **7a**	S (%) **8a**
CHP	93	0	59	14	0	0	0	0
TBHP $_{(dec.)}$	81	4	39	2	1	0	0	12
TBHP $_{(aq)}$	67	0	45	12	0	0	0	4
H_2O_2	71	0	4	11	17	7	4	0
No oxidant	11	0	0	0	0	0	0	0

Reaction conditions: styrene (10 mmol), oxidant (15 mmol), TBABr (10 mol%), $p\mathrm{CO_2}$ = 10 barg, T = 80 °C, t = 6 h, 600 rpm. Conversion and yields were determined by ^{1}H-NMR as described in the experimental section.

Table S3. Selectivity from Figure 4.

Pressure (barg)	X (%) **1a**	S (%) **2a**	S (%) **3a**
10	93	1	59
20	94	2	61
40	95	2	61
60	94	2	64

Reaction conditions: styrene (10 mmol), CHP (15 mmol), TBABr (10 mol %), $p\mathrm{CO_2}$ = see table, T = 80 °C, t = 6 h, 600 rpm. Conversion and yields were determined by ^{1}H-NMR as described in the experimental section.

Table S4. Selectivity from Figure 6.

Catalyst	X (%) **1a**	S (%) **2a**	S (%) **3a**	S (%) **4a**
CTABr	93	4	54	12
TBABr	93	1	59	13
TEABr	93	2	53	12
TMABr	85	44	16	11
TBACl	97	25	33	13
TBAOH*	100	51	8	19
bmimCl	98	24	34	15
bmimBr	93	3	55	14
PPNCl	89	3	46	15
PPNBr	91	0	63	13
No	58	27	0	5

Reaction conditions: styrene (10 mmol), CHP (15 mmol), catalyst (10 mol %), $p\mathrm{CO_2}$ = 10 barg, T = 80 °C, t = 6 h, 600 rpm. * TBAOH was used as 40 % wt aqueous solution in 3.75 mol% to keep the catalyst total loading (i.e. wt%) consistent and avoid detrimental cross-interaction with water. Conversion and yields were determined by ^{1}H-NMR as described in the experimental section of the main paper.

Table S5. Selectivity from Figure 8.

Run	X (%) **1a**	S (%) **2a**	S (%) **3a**	S (%) **4a**
First	93	1	59	13
Second*	92	5	52	13
Third*	92	7	51	12
Fourth*	90	8	50	12

Reaction conditions: styrene (10 mmol), CHP (15 mmol), catalyst (10 mol %), pCO_2 = see table, T = 80 °C, t = 6 h, 600 rpm. * The TBABr recovered by water extraction was employed. Conversion and yields were determined by ^{1}H-NMR as described in the experimental section.

Table S6. Selectivity from Figure S4.

Entry	Cat. load. [mol%]	X (%) **1a**	S (%) **2a**	S (%) 3**a**
1	1	27	63	7
2	5	46	33	39
3	10	70	14	59

Reaction conditions: styrene (10 mmol), CHP (15 mmol), catalyst (see table), pCO_2 = 10 barg, T = 80 °C, t = 6 h, 600 rpm.

5. Influence of CHP on the carbonation reaction

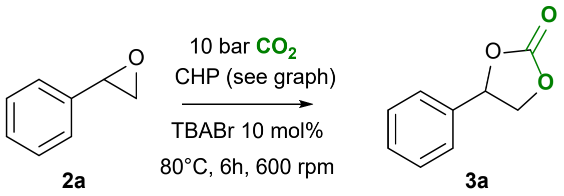

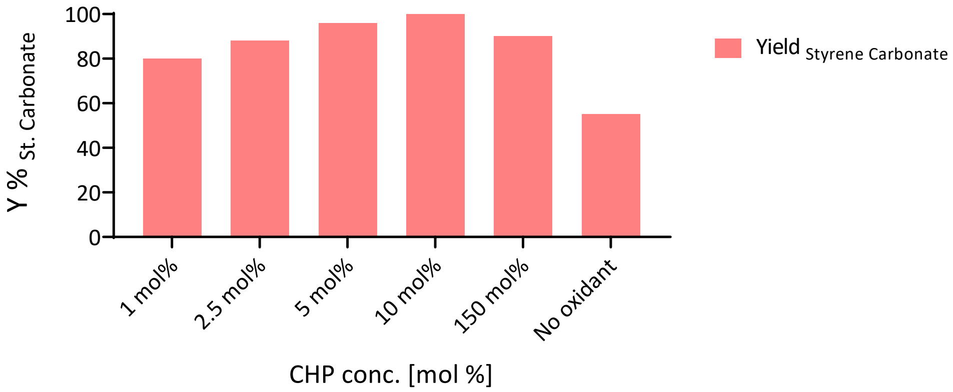

Figure S7. Effect of the oxidant in the carbonation step studied using styrene oxide **2a** as a starting material. In all cases, the selectivity was >95%, apart from the test with 150 mol% in which the selectivity was 90%. Reaction conditions: styrene oxide (20 mmol), CHP 80 wt% (see graph for the loading), tetrabutylammonium bromide (10 mol%), pCO_2 = 10 barg, T = 80 °C, and 600 rpm.

6. Performance of different tetra-alkyl substituted bromide salts

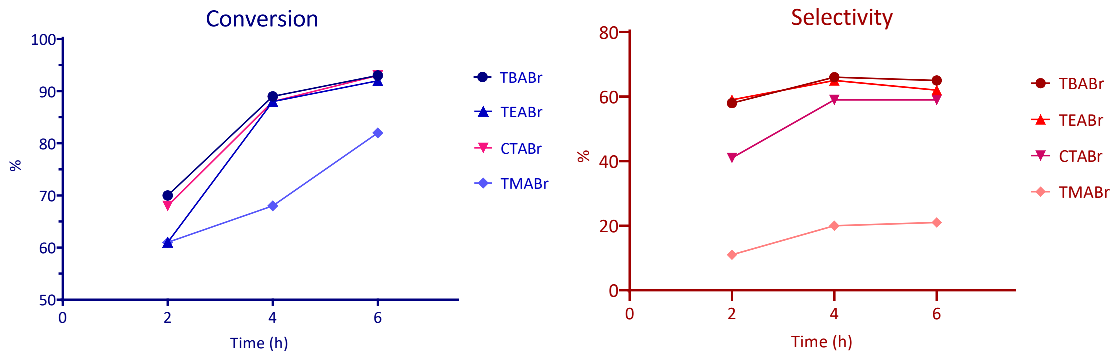

Figure S8. Comparison of conversion (left) and selectivity (right) of different tetralkyl-substituted ammonium bromide salts at different reaction times for styrene carbonate, **3a**.

7. ^{1}H-NMR and GC-MS analysis of the crude mixture for the 3 h synthesis

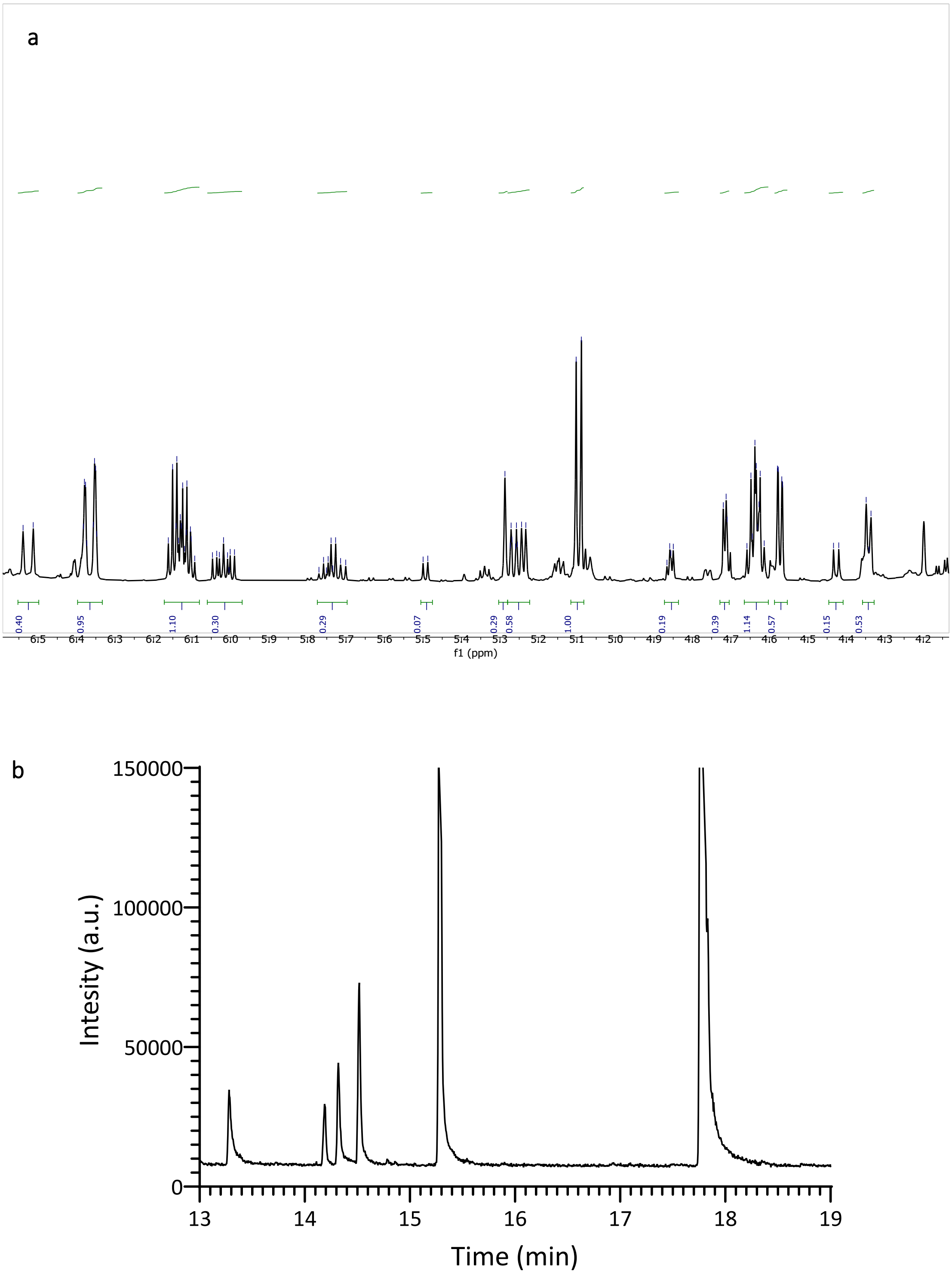

Figure S9. (a) ^{1}H-NMR spectrum of the reaction mixture obtained according to the experimental procedure for the synthesis of **3h**. Several peaks in the region 4.00-6.00 ppm related to oxidation by-products are present (see Figure S13h for the ^{1}H-NMR spectrum of the isolated compound). (b) Gas Chromatogram (t_R 13.00-19.00 min) of the fraction obtained after the first purification step (see section 8 of SI). GC-MS (ESI) [t_R (min), m^+/z]: (a) 13.30, 194; (b) 14.20, 194; (c) 14.35, 208; (d) 14.55, 210; (e) 15.30, 210; (f) 17.90, 238.

8. TBABr and CHP side-reactions

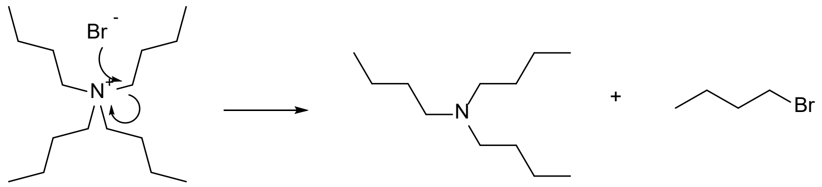

Figure S10. Tetrabutylammonium decomposition mechanism through the Intramolecular nucleophilic substitution leading to tributylamine and butyl-bromide.

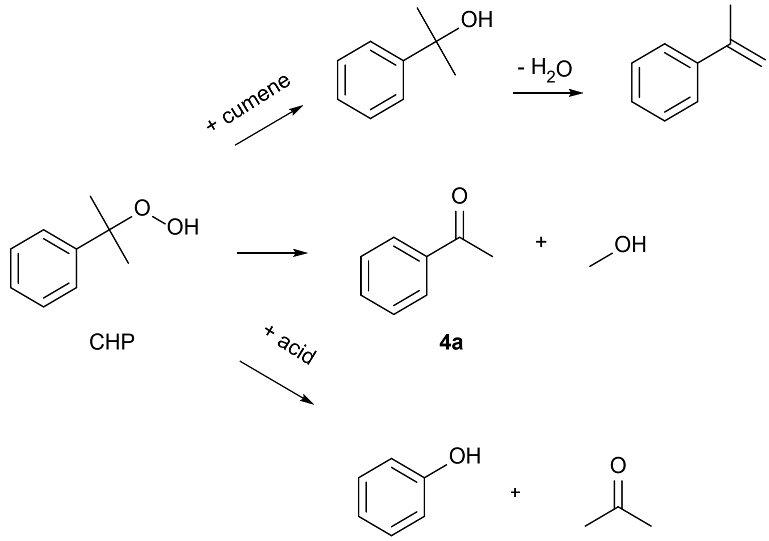

Figure S11. Possible side reactions of cumene hydroperoxide reported by Levin et al.[6] The reaction conditions of the control experiments were: CHP (15 mmol), pCO_2 = 10 bar, T = 80 °C, t = 6 h, 600 rpm. Conversion and yields were determined by ^{1}H-NMR and based on the initial amount of CHP (methyl groups were used for the integration).

9. ^{1}H-NMR-spectra of relevant compounds and reaction mixtures

The assignments of the peaks in the ^{1}H-NMR spectra of the epoxides **2a-j** and of the cyclic carbonates **3a-j** were based on data from the literature.[7–10] Such data are not available for the compounds obtained from methylisoeugenol. Therefore, the cyclic carbonate **3h** prepared from methylisoeugenol was isolated (see section 10).

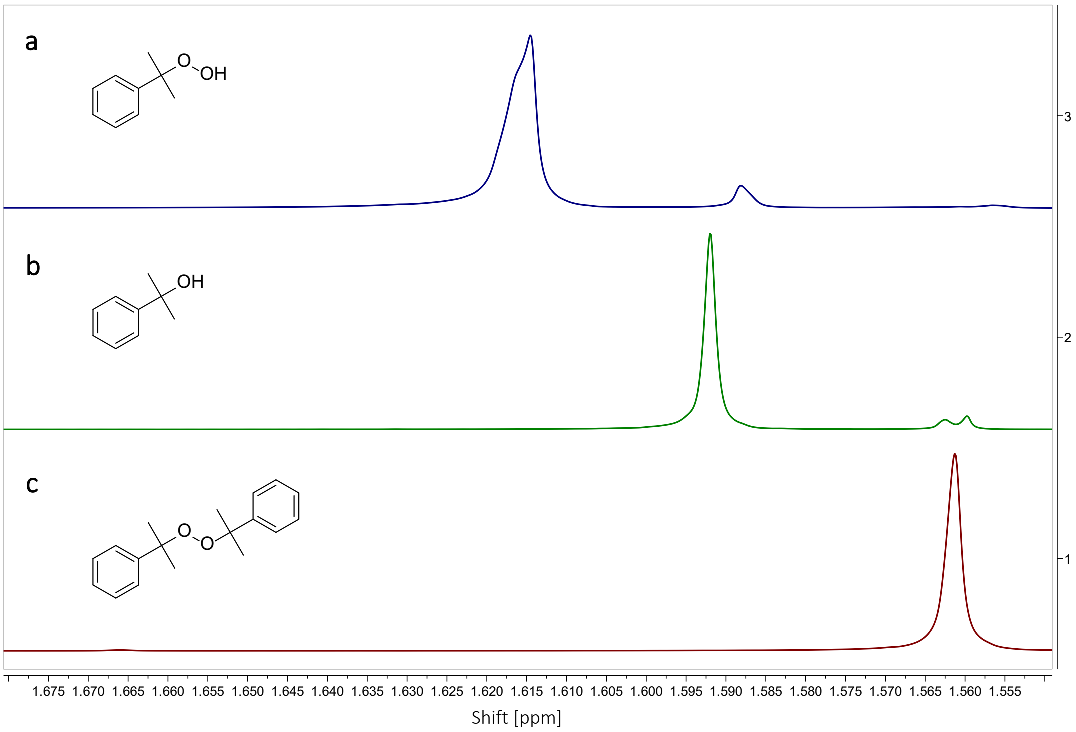

Figure S12. 1.680-1.550 ppm region of the ^{1}H-NMR spectrum of the pure compounds recorded at 25 °C, showing the peaks corresponding to the methyl groups of: (a) CHP, (b) 2P2P, and (c) DCP.

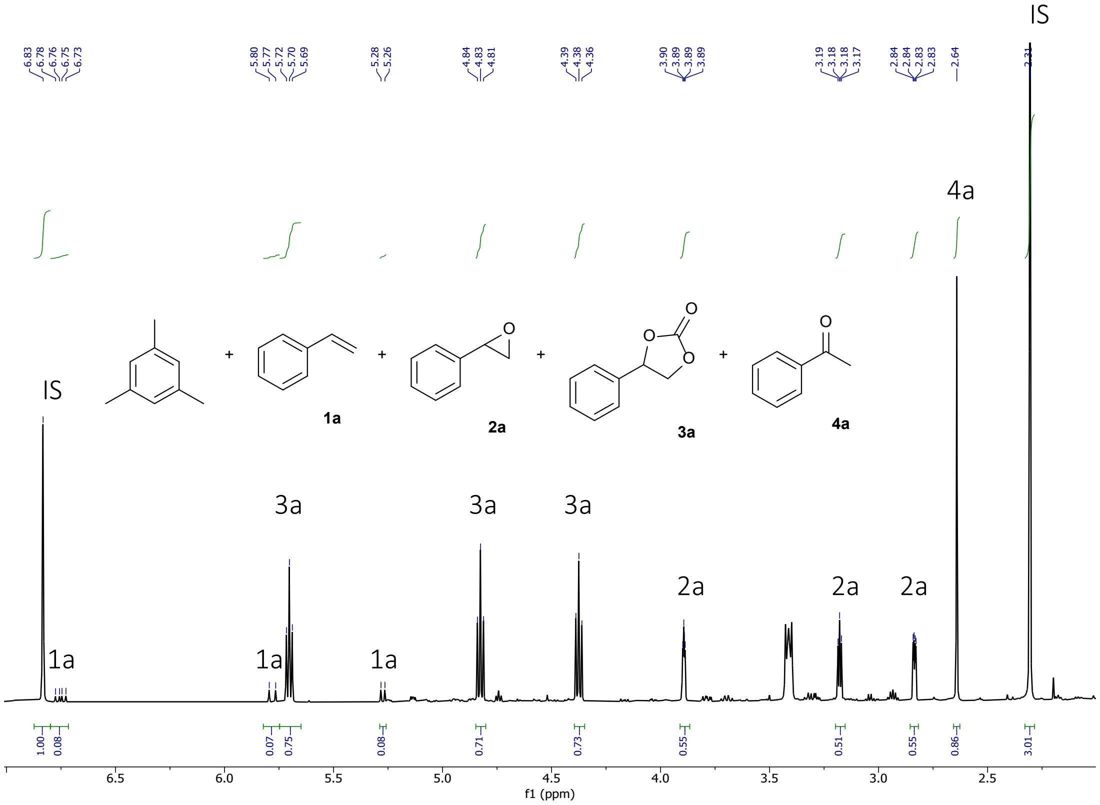

Figure S13a. ^{1}H-NMR spectrum of the reaction mixture after the synthesis of **2a** and **3a** from **1a** with TBACl as catalyst. Reaction conditions and results are reported in Figure 6. The peaks used for quantification in a typical experiment are reported. IS: internal standard (mesitylene). **^{1}H NMR (600 MHz, $CDCl_3$)**: 5.94-5.91 (t, 1H), 5.68-5.65 (d, 1H), 5.33-5.31 (d, 1H), 4.87-4.84 (t, 1H), 4.32-4.29 (t, 1H), 4.04-4.02 (m, 1H), 3.31-3.27 (m, 1H), 2.73-2.71 (m, 1H).

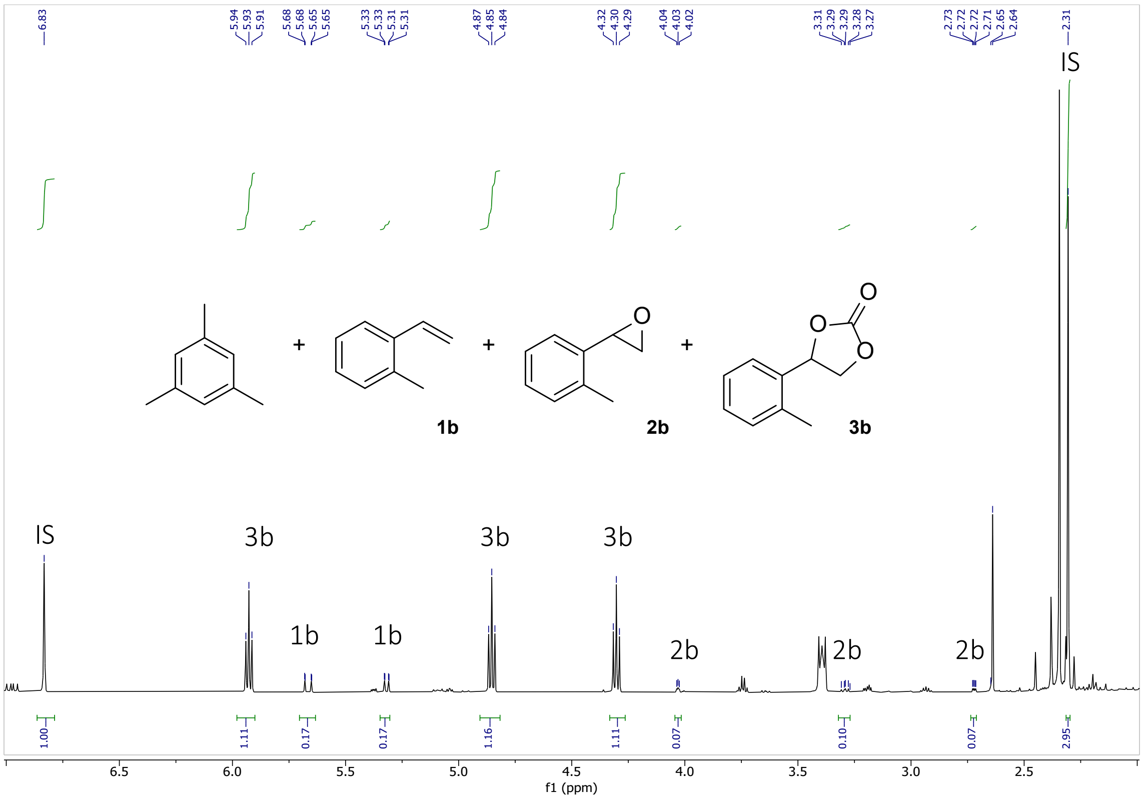

Figure S13b. ^{1}H-NMR spectrum of the reaction mixture after the synthesis of **2b** and **3b** from **1b**. Reaction conditions and results are reported in Figure 7. **^{1}H NMR (600 MHz, $CDCl_3$)**: 5.94-5.91 (t, 1H), 5.68-5.65 (d, 1H), 5.33-5.31 (d, 1H), 4.87-4.84 (t, 1H), 4.32-4.29 (t, 1H), 4.04-4.02 (m, 1H), 3.31-3.27 (m, 1H), 2.73-2.71 (m, 1H).

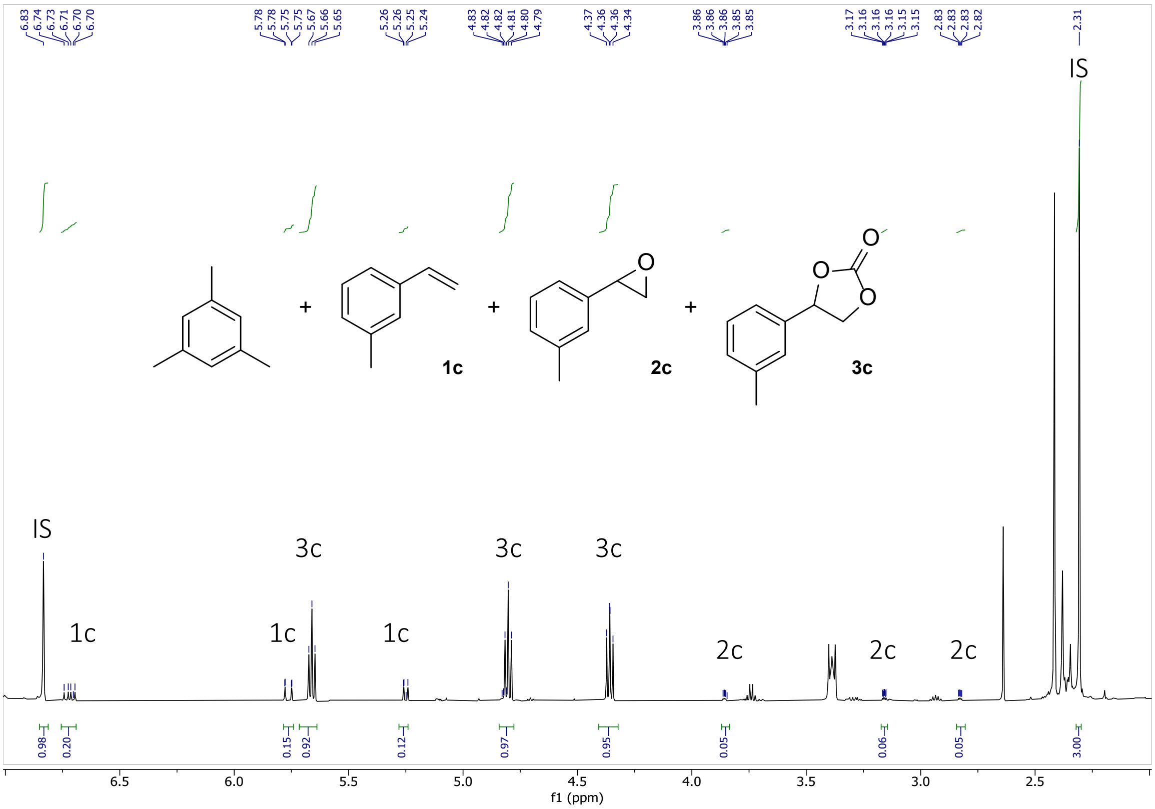

Figure S13c. ^{1}H-NMR spectrum of the reaction mixture after the synthesis of **2c** and **3c** from **1c**. Reaction conditions and results are reported in Figure 7. **^{1}H NMR (600 MHz, $CDCl_3$)**: 6.73-6.70 (dd, 1H), 5.78-5.75 (d, 1H), 5.67-5.65 (t, 1H), 5.26-5.24 (d, 1H), 4.83-4.79 (t, 1H), 4.37-4.34 (t, 1H), 3.86-3.85 (m, 1H), 3.17-3.15 (m, 1H), 2.83-2.82 (m, 1H).

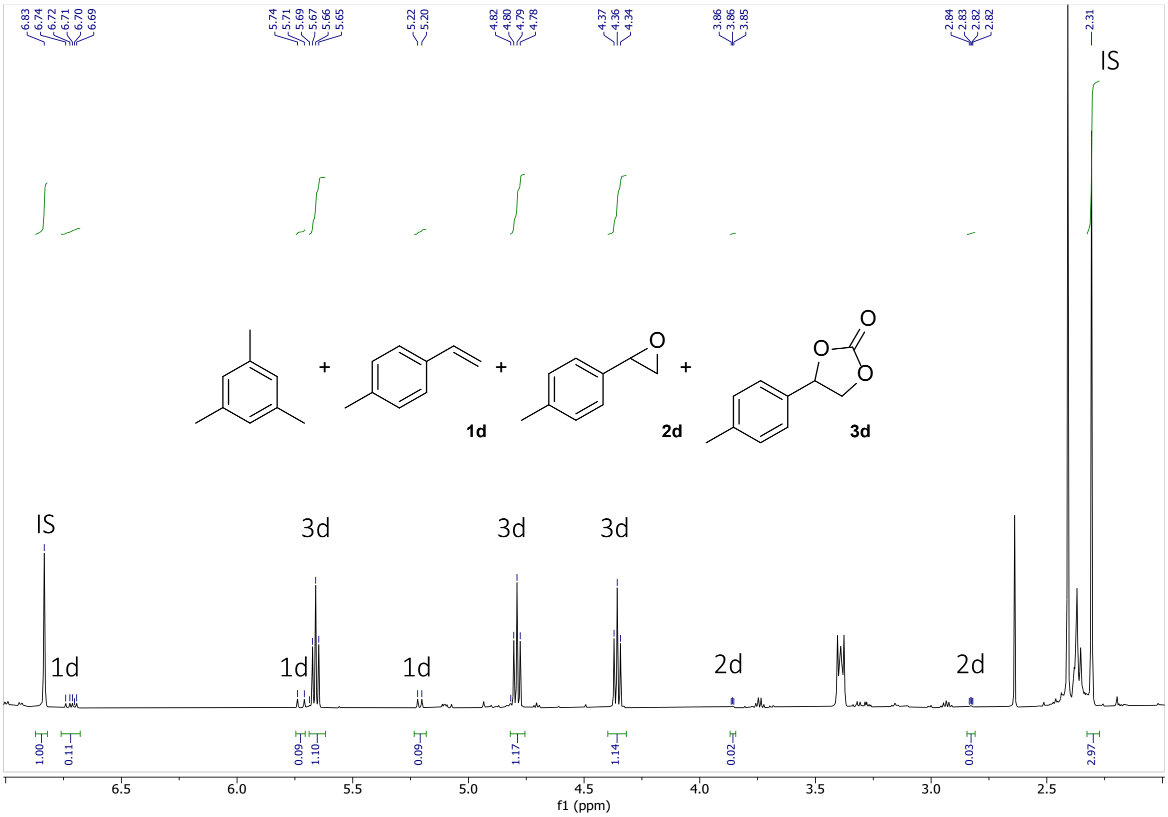

Figure S13d. ^{1}H-NMR spectrum of the reaction mixture after the synthesis of **2d** and **3d** from **1d**. Reaction conditions and results are reported in Figure 7. **^{1}H NMR (600 MHz, $CDCl_3$)**: 6.74-6.69 (dd, 1H), 5.74-5.71 (d, 1H), 5.69-5.65 (t, 1H), 5.22-5.20 (d, 1H), 4.82-4.78 (t, 1H), 4.37-4.34 (t, 1H), 3.86-3.84 (m, 1H), 2.84-2.82 (m, 1H).

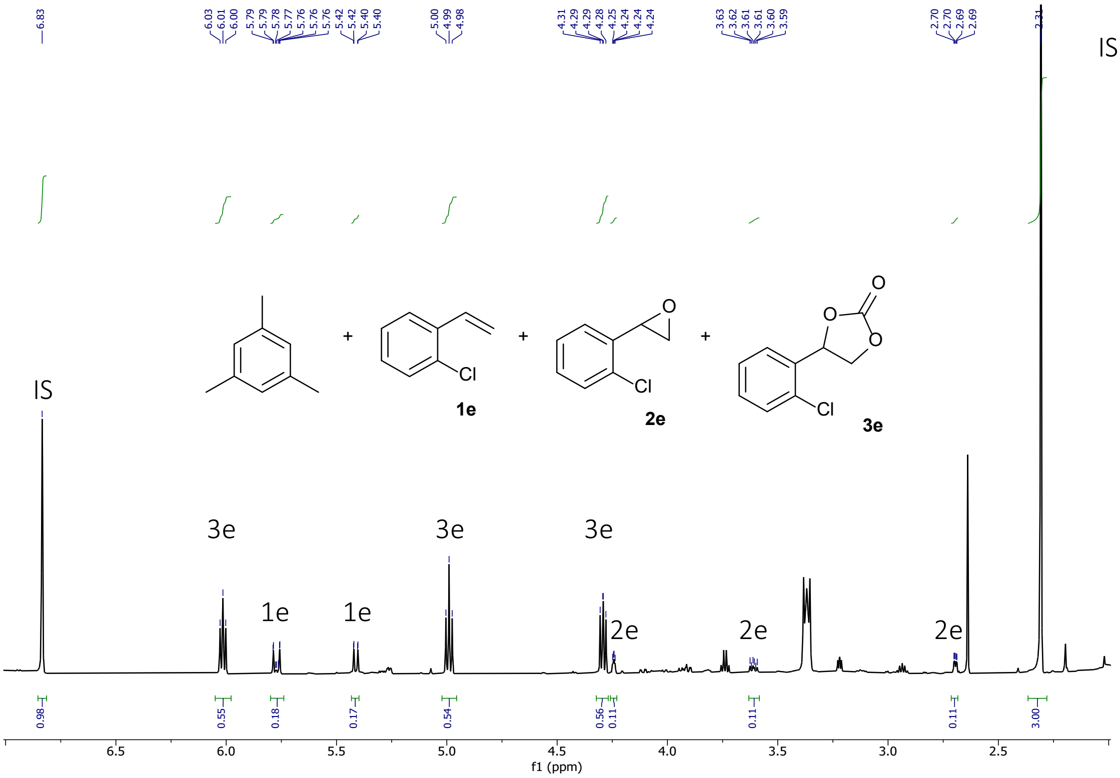

Figure S13e. ^{1}H-NMR spectrum of the reaction mixture after the synthesis of **2e** and **3e** from **1e**. Reaction conditions and results are reported in Figure 7. **^{1}H NMR (600 MHz, $CDCl_3$)**: 6.03-6.00 (t, 1H), 5.79-5.76 (m, 1H), 5.42-5.40 (d, 1H), 5.00-4.98 (t, 1H), 4.31-4.28 (t, 1H), 4.25-4.24 (m, 1H), 3.61-3.59 (m, 1H), 2.70-2.64 (m, 1H).

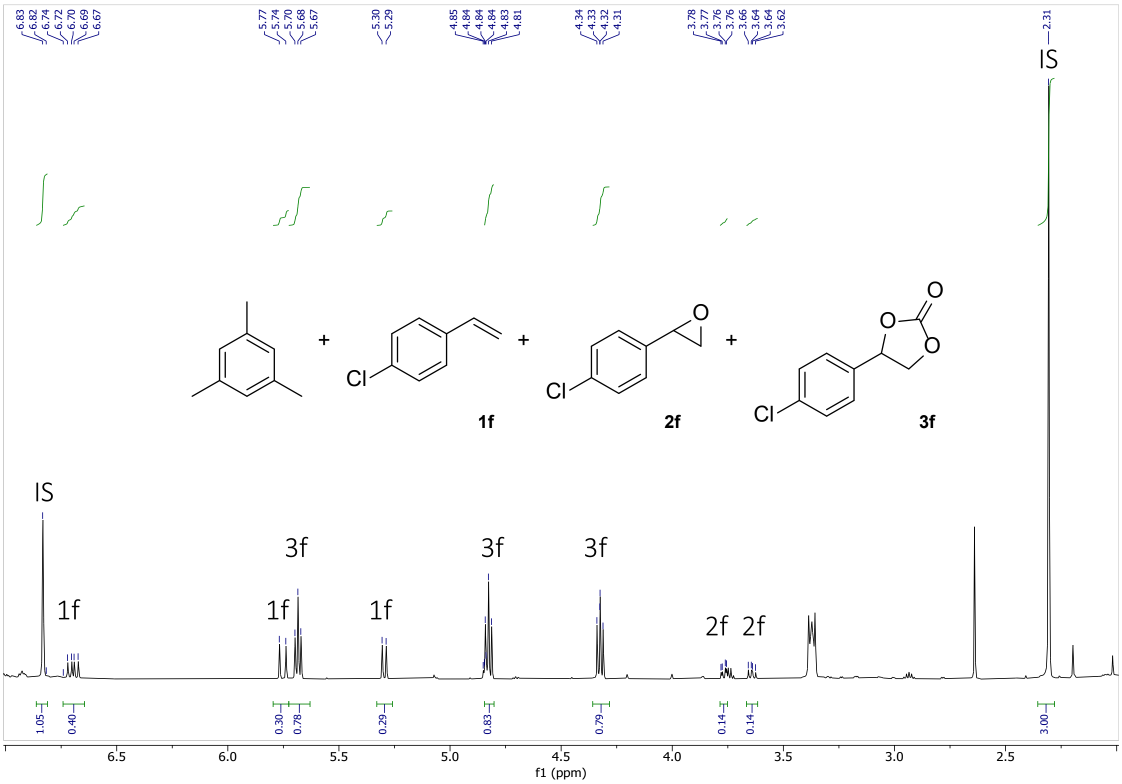

Figure S13f. ^{1}H-NMR spectrum of the reaction mixture after the synthesis of **2f** and **3f** from **1f**. Reaction conditions and results are reported in Figure 7.. **^{1}H NMR (600 MHz, $CDCl_3$)**: 6.74-6.87 (dd, 1H), 5.77-5.74 (d, 1H), 5.70-5.67 (t, 1H), 5.30-5.29 (d, 1H), 4.85-4.81 (t, 1H), 4.34-4.31 (t, 1H), 3.78-3.71 (dd, 1H), 3.65-3.62 (dd, 1H).

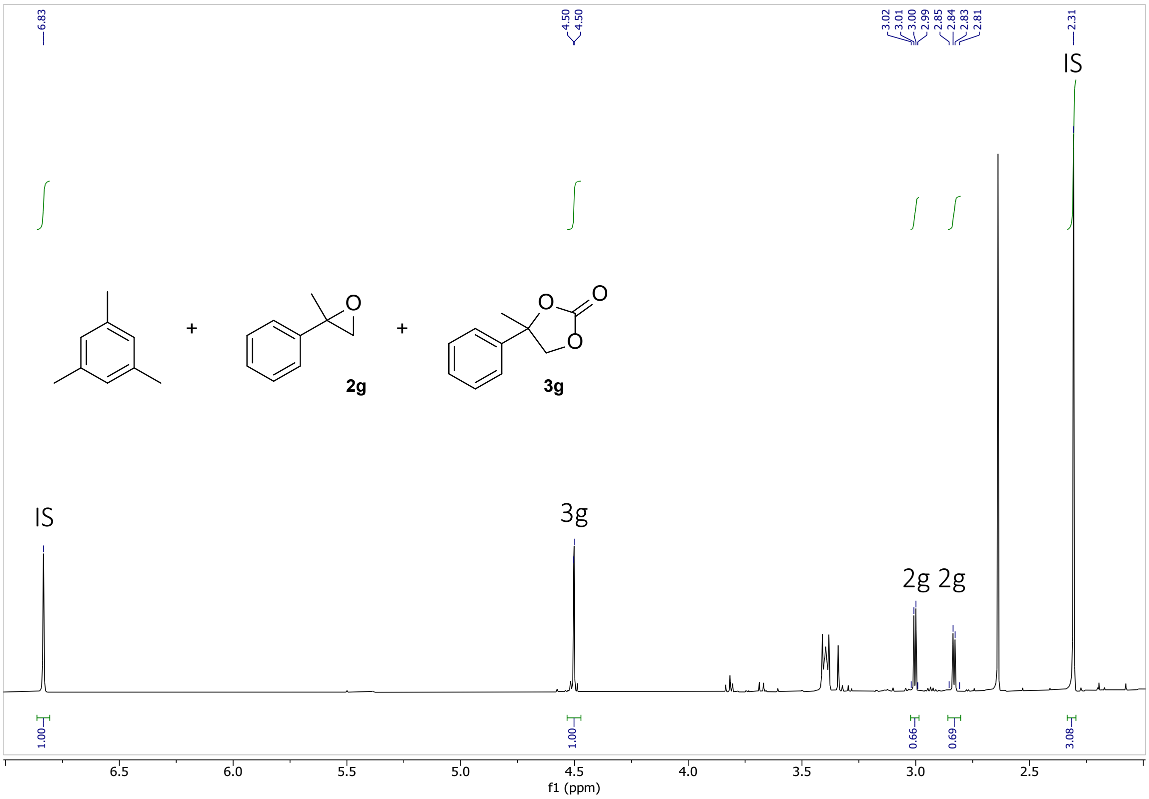

Figure S13g. ^{1}H-NMR spectrum of the reaction mixture after the synthesis of **2g** and **3g** from **1g** (48 hours). Reaction conditions and results are reported in Figure 7. **^{1}H NMR (600 MHz, CDCl$_3$)**: 4.50 (s, 2H), 3.02-2.99 (d, 1H), 2.85-2.81 (d, 1H).

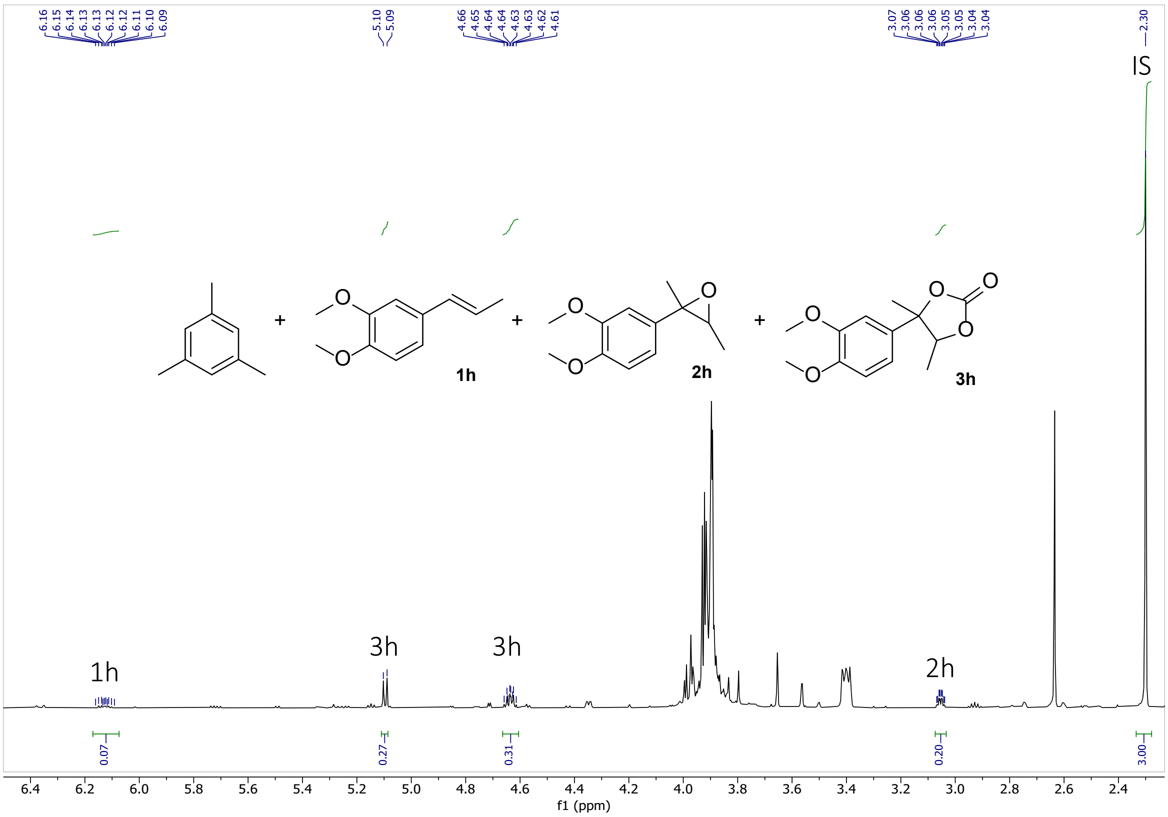

Figure S13h (a). ^{1}H-NMR spectrum of the reaction mixture after the synthesis of **2h** and **3h** from **1h** (18 hours). Reaction conditions and results are reported in Figure 7. **^{1}H NMR (600 MHz, CDCl$_3$)**: 6.16-6.09 (m, 1H), 5.10-5.09 (d, 1H), 4.65-4.61 (dq, 1H), 3.07-3.04 (qd, 1H). The product **2h** was assigned through computational methods whereas the target product **3h** was isolated (see section 10 of the ESI).

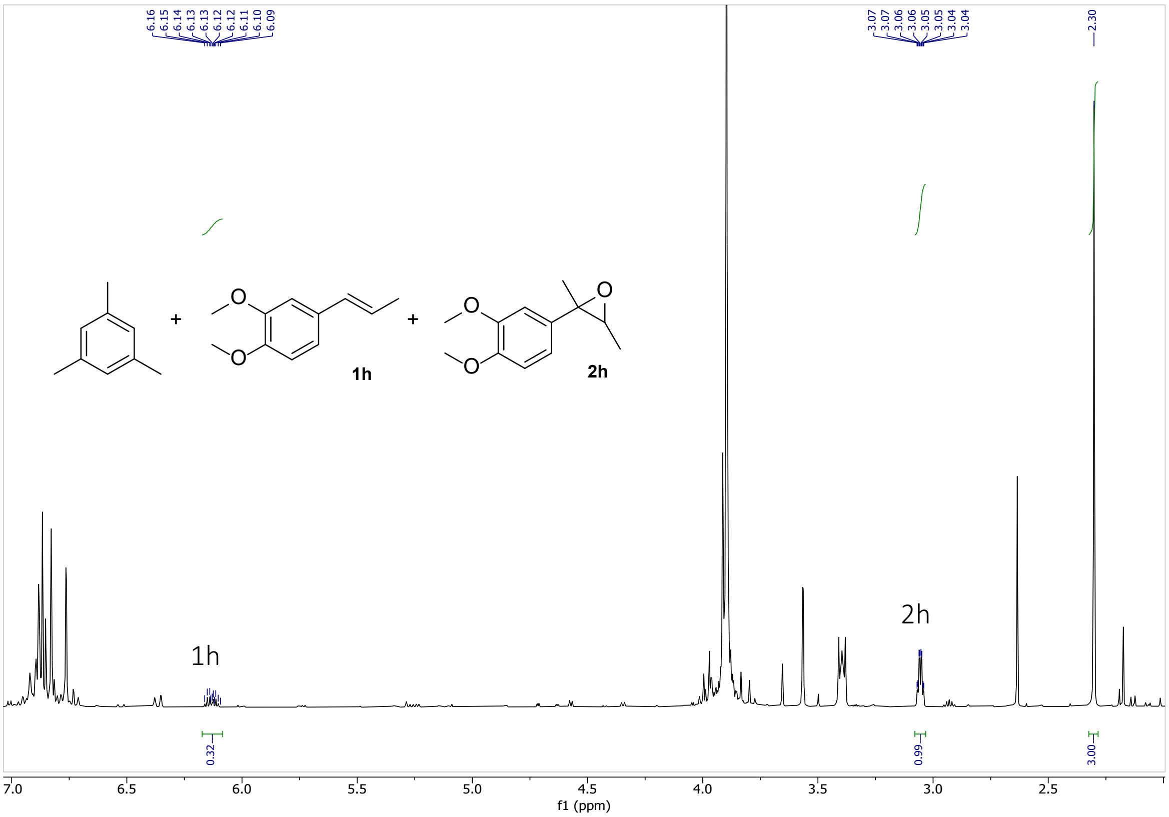

Figure S13h (b). ^{1}H-NMR spectrum of the reaction mixture after the synthesis of **2h** and **3h** from **1h** (6 hours, no CO_2). Reaction conditions and results are reported in Figure 7. **^{1}H NMR (600 MHz, $CDCl_3$)**: 6.16-6.09 (m, 1H), 3.07-3.04 (qd, 1H).

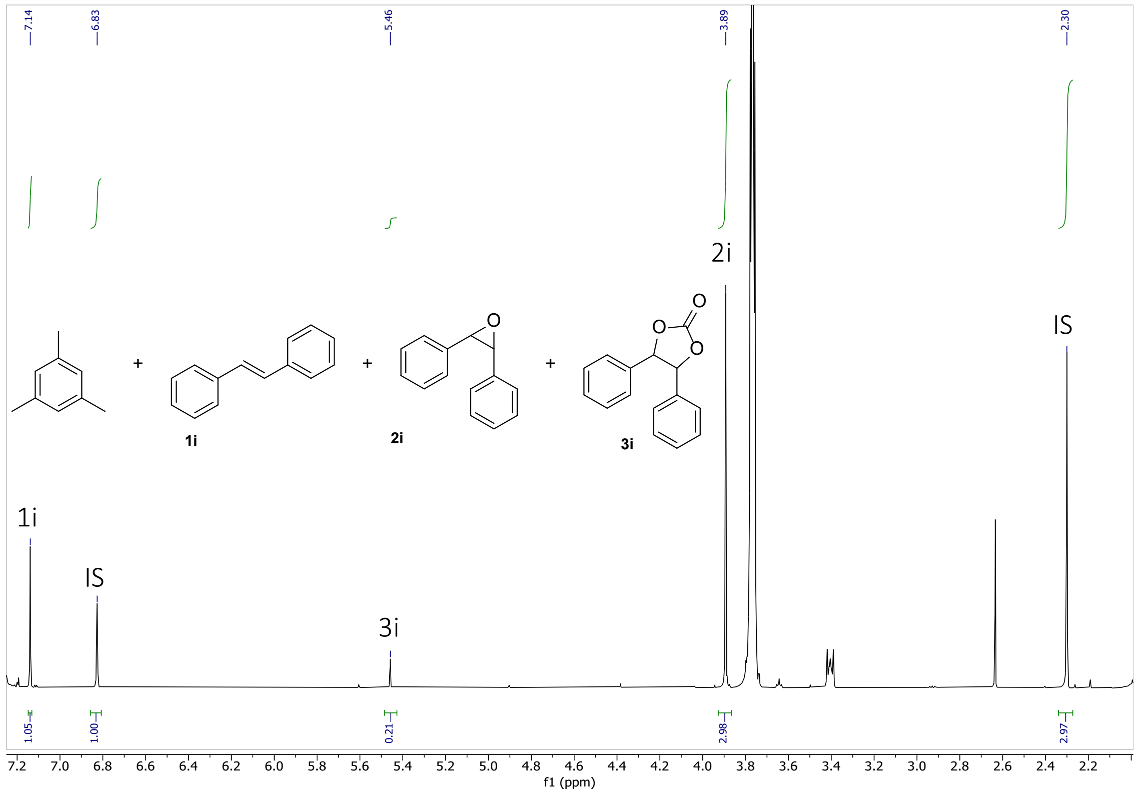

Figure S13i. ^{1}H-NMR spectrum of the reaction mixture after the synthesis of **2i** and **3i** from **1i**. Reaction conditions and results are reported in Figure 7. **^{1}H NMR (600 MHz, $CDCl_3$)**: 7.14 (s, 2H), 5.49 (s, 2H), 3.89 (s, 2H). The large peak at 3.75 ppm derives from residual THF.

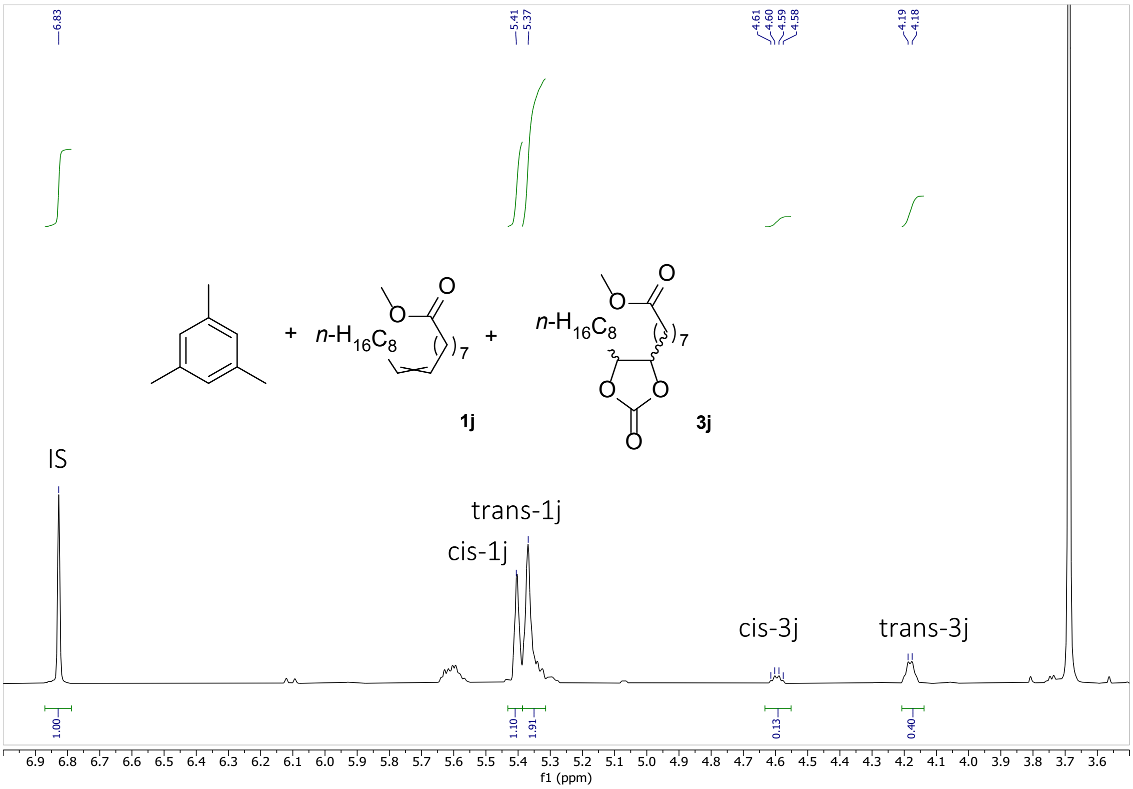

Figure S13j. ^{1}H-NMR (7.00-3.00 ppm) of the reaction mixture after the synthesis of **2j** and **3j** from **1j**. Reaction conditions and results are reported in Figure 7. **^{1}H NMR (600 MHz, $CDCl_3$)**: 5.41 (s, 2H), 5.37 (s, 2H), 4.61-4.58 (q, 2H), 4.19-4.18 (m, 2H). Based on the integration of the peaks, the following ratios were determined: 1.7:1.0 for trans:cis methyl oleate and 3.1:1.0 for trans:cis carbonate. The isomerisation of the starting **1j** is attributed to the relatively high temperature (80 °C), combined with the presence of radicals generated by CHP. Moreover, in accordance with literature, the cycloaddition is not regioselective leading to both cis- and trans-carbonate.[7]

10. Isolation and NMR spectra of methylisoeugenol carbonate, 3h

Isolation of 4-(3,4-dimethoxyphenyl)-5-methyl-1,3-dioxolan-2-one or methylisoeugenol carbonate, 3h

The cyclic carbonate **3h** was obtained starting from the corresponding starting material **1h** following the procedure described in the experimental section using 18 h as reaction time. The isolation of **3h** was done through two consecutive separation steps. Initially, the reaction mixture was deposited on solid silica and the product (R_f = 0.18 in hexane:ethyl acetate = 70:30, v:v) was collected by column chromatography using hexane:ethyl acetate with gradient ranging from 100:0 to 50:50 as mobile phase. The gradient was adjusted by 5:5 every 50 mL of used mobile phase and p-anisaldehyde staining solution was used for TLC [11]. The product was collected and water was removed by means of $MgSO_4$. The solid residue was removed by filtering the solution, and the solvent was evaporated in a rotary evaporator. Due to co-elution (see Figure S9b), the product was further purified by means of reverse-phase preparative HPLC using LC-20AD prominence equipped with SPD-M20A prominence diode array detector, an Xselect®CSH C18 OBD™ Prep column (130 Å, 5 µm, 10 mm x 250 mm, 1/pkg) and controlled by Shimadzu software. A solution of the partially purified crude mixture was prepared by dissolving 200 mg of it in 1.2 mL of a mixture acetonitrile:water (60:40, v:v) into an HPLC glass vial sealed with an aluminium crimp-cap. Acetonitrile (HPLC grade, Sigma) + 0.1 wt% formic acid (Solvent A) and water + 0.1 wt% formic acid (Solvent B) were used as mobile phase in different ratios. In the reported conditions, the product showed a t_R = 25.90 min and the following set points were used: 5.00 min (A:B 60:40, v:v), 20.00 min (A:B 95:5, v:v), 21.00 min (A:B 5:95, v:v), 26.00 min (A:B 5:95, v:v), 30.00 min (ending time, A:B 5:95, v:v). The injection volume was 100 µL and the column oven was kept at 40 °C. The solvent was removed by rotary evaporation under reduced pressure and dried at 4 mbar overnight using a vacuum pump, and finally the product was recovered.

4-(3,4-dimethoxyphenyl)-5-methyl-1,3-dioxolan-2-one or methylisoeugenol carbonate, 3h. White solid. **^{1}H-NMR (600 MHz, $CDCl_3$)**: δ 6.88 (m, 3H), 5.07 (d, J = 8.2 Hz, 1H), 4.61 (dq, J = 8.2, 6.2 Hz, 1H), 3.91 (s, 1H), 3.90 (s, 1H), 1.54 (d, J = 6.2 Hz, 3H). **^{13}C-NMR (151 MHz, $CDCl_3$)**: δ 154.23, 150.27, 149.71, 127.08, 119.21, 111.23, 108.73, 85.20, 80.69, 56.10, 56.03, 18.23.

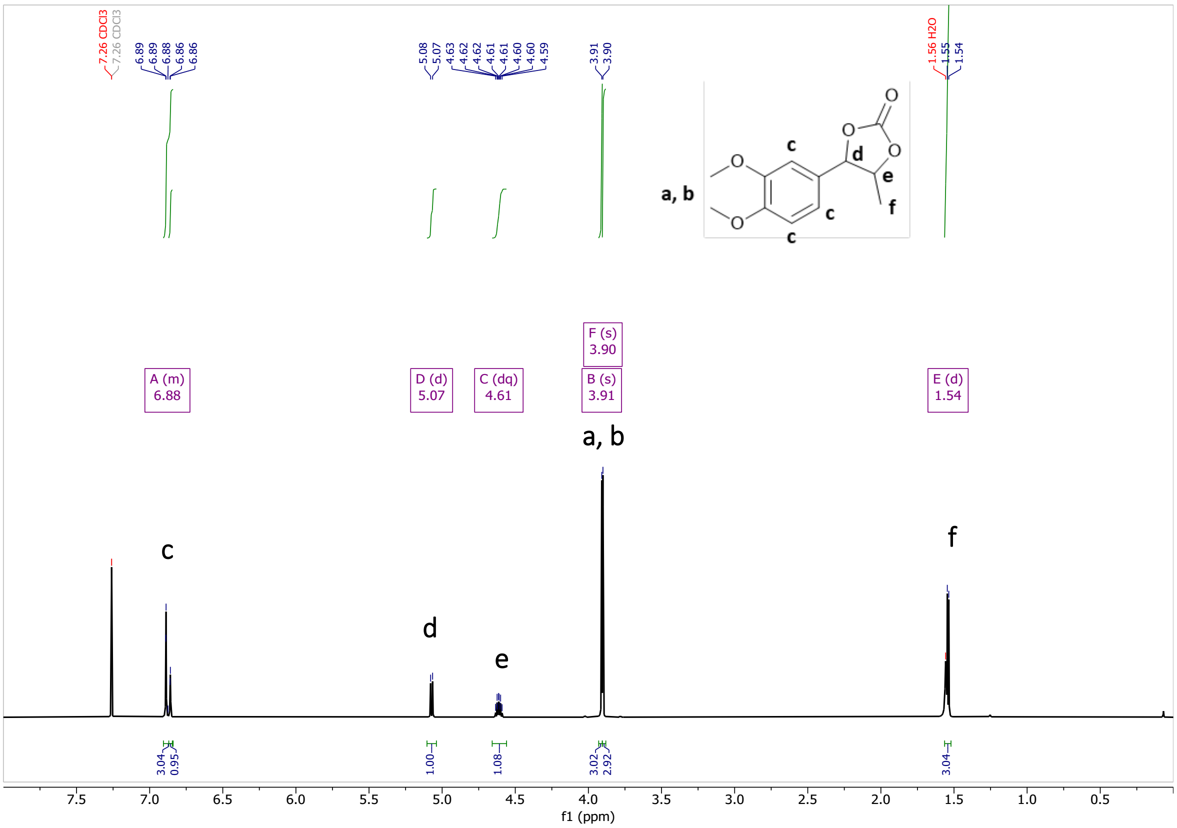

Figure S14. ^{1}H-NMR spectrum of the isolated **3h.**

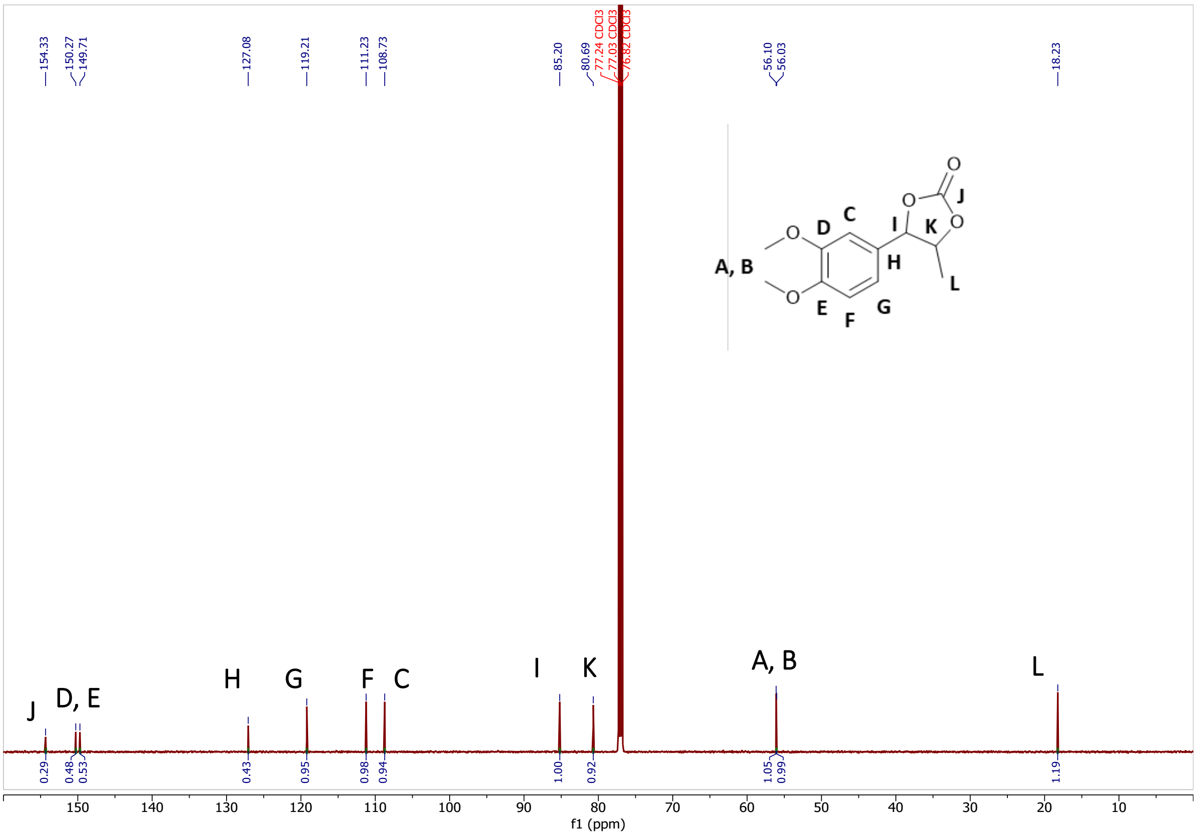

Figure S15. ^{13}C-NMR spectrum of the isolated **3h.**

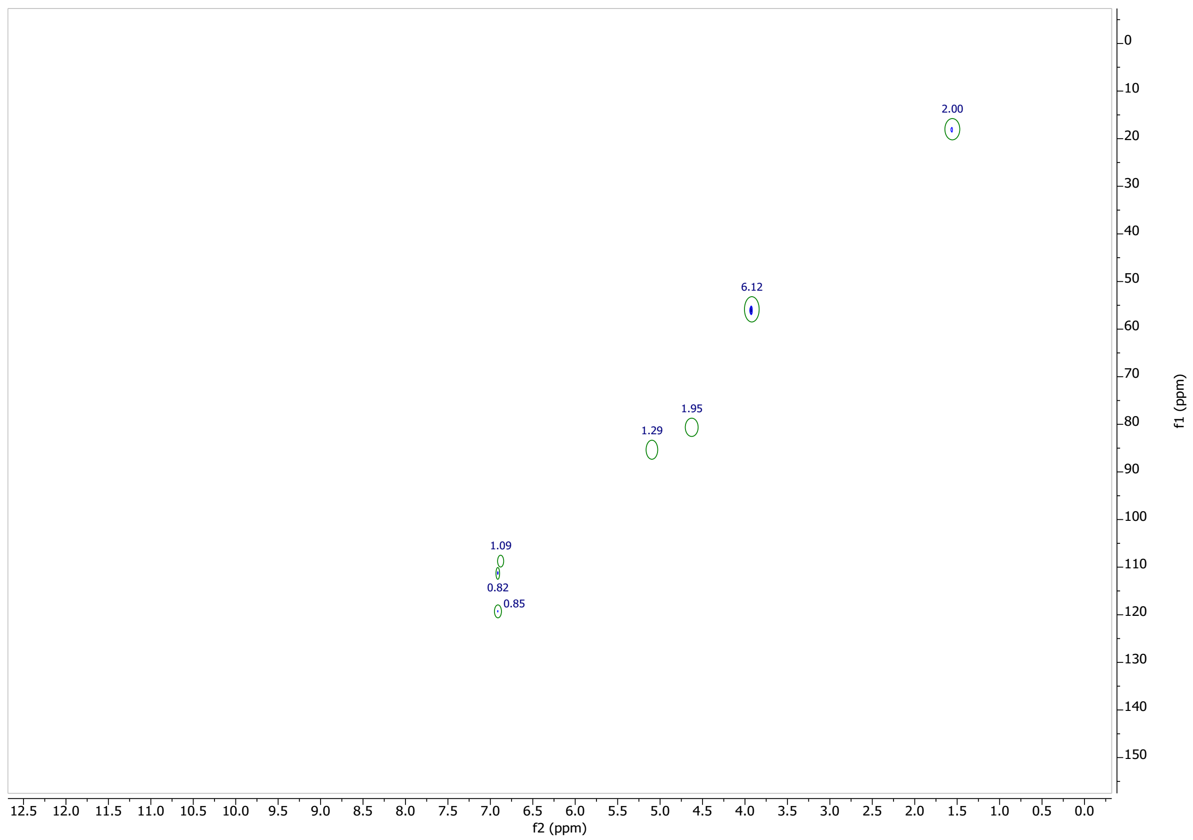

Figure S16. HSQC-NMR spectrum of the isolated **3h.**

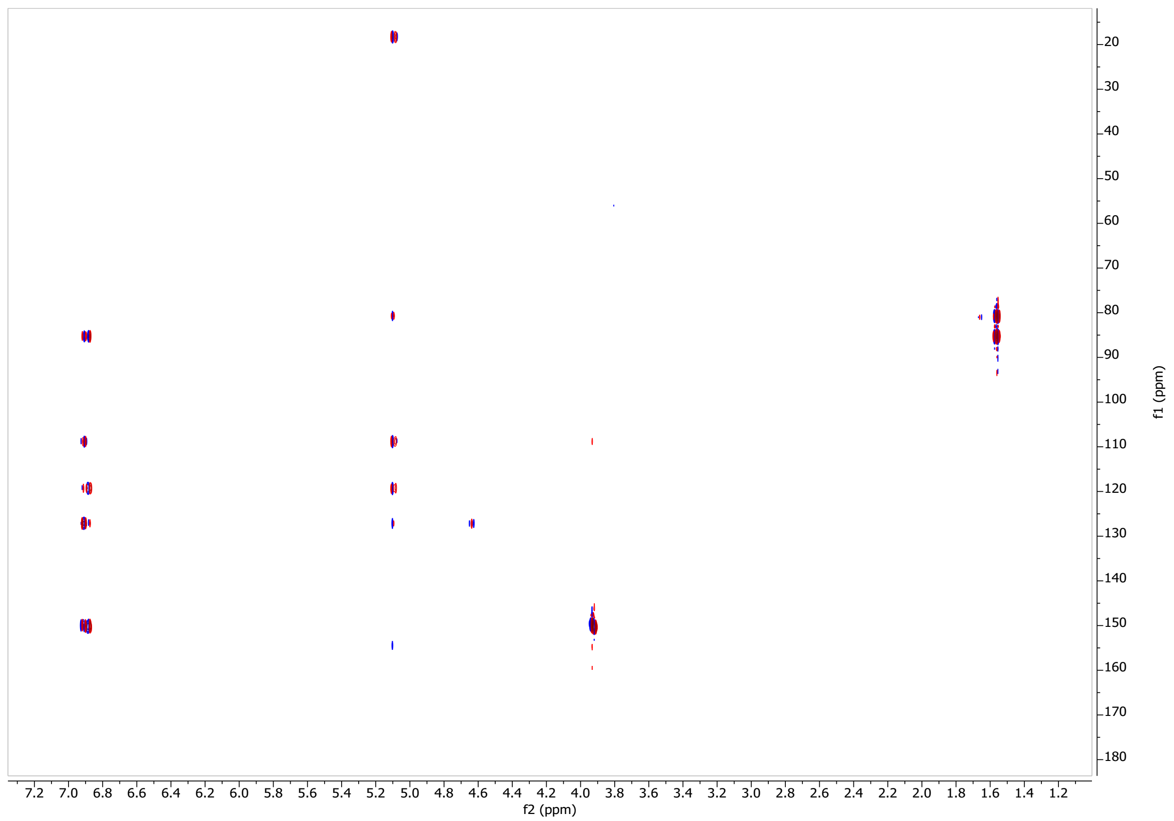

Figure S17. HMBC-NMR spectrum of the isolated **3h**.

11. Reproducibility

To evaluate the reproducibility of our results, we performed three test in absence of catalyst and under N_2 atmosphere and two times under CO_2 pressure in presence of 10 mol% of the TBABr following the procedure described in the experimental section. The results, the mean and the standard deviations are reported in Table S7. The tests demonstrated good reproducibility. Therefore, no further repetitions of the experiments were systematically conducted in this work.

Table S7. Reproducibility results.

Entry	Gas	Conv. [%]	Mean ± Std. Dev.	Y_{SO} [%]	Mean ± Std. Dev.	Y_{SC} [%]	Mean ± Std. Dev.
1 [a]	N_2	52	55 ± 2	17	16 ± 1	-	-
2 [a]		58		17		-	
3 [a]		56		14		-	
4	CO_2	93	-	1	-	55	-
5		93		1		55	

Reaction conditions: styrene (10 mmol), CHP 80 wt% 1.5 equiv, 10 mol % TBABr, p = 10 barg, T = 80 °C, t = 6 h. [a] No TBABr. Conversion, yields and mole balance were determined by ^{1}H-NMR as described in the experimental section.

12. Comparison with state-of-the-art catalytic systems in the literature

Table S8. Comparison of the state-of-the-art non-assisted one-pot processes utilising one oxidant and a single catalyst.

Entry [a]	Oxidant		Catalyst	Solv.	pCO_2 [bar]	T [°C]	t [h]	X [%]	Y_{3a} [%]	S_{3a} [%]	Ref.
1	CHP 80%	1.5 eq.	TBABr 10 mol %	-	10	80	6	93	55	59	this work
2	CHP 80%	4 eq.	TBABr 10 mol %	-	10	80	6	99	67	67	this work
3	$TBHP_{(aq)}$	1.5 eq.	TBABr 10 mol %	-	10	80	6	67	30	45	this work
4	O_2 [b,c]	8 bar	TBABr 8 mol %	AcN [c]	45	130	7	99	61	61	[12]
5	$TBHP_{(aq)}$	1.5 eq.	TBABr 8 mol%	-	10	80	6	94	38	40	[13]
6	TBHP [d]	2 eq.	$[C_1C_4Im][HCO_3]$ 6 mol%	-	20	50	30	56	50	89 [f]	[14]
7	$TBHP_{(aq)}$	3 eq.	$[Im][HCO_3]$@SBA15 50 mg/mmol [e]	AcN	10	80	28	95	82	88 [f]	[15]

[a] All the entries compare the performance based on styrene **1a** as the substrate.

[b] Introduced as synthetic air.

[c] The substrate concentration in acetonitrile is 35 mM and 5 equiv of isobutyraldehyde (IBA) are used as sacrificial species.

[d] The authors did not specify whether the TBHP is aqueous or in organic solvent.

[e] Heterogeneous bifunctional catalyst.

[f] In our work, we observed polystyrene formation in the presence of CHP or TBHP and accounted for it in the calculation of the selectivity, whereas in these two works the formation of the polymer was not addressed.

13. DFT geometries (xyz)

CO_2 (3 atoms)

C	-0.62543653	-0.98931619	0.00459839
O	-1.84344956	-0.91971620	0.00427488
O	0.59456347	-0.98931619	0.00459839

Styrene, **1a** (16 atoms)

C	-0.42004441	1.72929695	0.01770685
C	-1.54822332	0.90406633	0.00787709
C	-1.40083664	-0.48356295	0.03009854
C	-0.12468403	-1.04842405	0.06165311
C	1.00865371	-0.22845720	0.07123268
C	0.87323392	1.17675933	0.05049271
H	-0.55804212	2.80361419	-0.00173159
H	-2.53776634	1.34187454	-0.01859362
H	-2.27553866	-1.12096790	0.02253812
H	-0.01231567	-2.12476530	0.07961158
H	1.97557736	-0.70741688	0.09255436
C	2.03610465	2.10727928	0.05522826
C	3.31983735	1.73027735	0.12018246
H	3.62995139	0.69555273	0.17864201
H	4.09822028	2.48734088	0.11817002
H	1.83594901	3.17432514	0.00565204

Styrene oxide, **2a** (17 atoms)

C	-0.66983631	2.46251835	-0.44114555
C	-1.83485766	1.76312431	-0.10890166
C	-1.77575626	0.39456212	0.16835018
C	-0.55347977	-0.27980123	0.10201906
C	0.61516501	0.41716249	-0.22295014
C	0.56637936	1.79717701	-0.47793712
C	1.82484614	2.54514693	-0.81226914
C	2.81551274	2.65457767	0.28474624

O	1.96042837	3.72210654	-0.04898191
H	-0.72958107	3.52210666	-0.65707550
H	-2.78316739	2.28253900	-0.06398785
H	-2.67680582	-0.14393611	0.43151273
H	-0.51226940	-1.34208567	0.30502364
H	1.55670244	-0.11597589	-0.27359198
H	2.16890529	2.67442527	-1.85968750
H	3.86154291	2.87117050	-0.01591054
H	2.54661687	2.22879626	1.27525692

Styrene carbonate, **3a** (20 atoms)

C	-0.42226632	2.35985341	-0.15664398
C	-1.68193731	1.87700285	-0.52642264
C	-1.95633875	0.50890436	-0.46064737
C	-0.97183183	-0.38015360	-0.02363349
C	0.28738700	0.09716728	0.35211496
C	0.57899464	1.47173843	0.28704898
C	1.95162665	1.95720422	0.71324130
O	3.58397471	4.71015819	-0.23654359
C	3.06209777	1.41863069	-0.19143379
O	3.86043127	2.50082029	-0.56128480
C	3.18187633	3.57425264	-0.04689588
O	2.04267884	3.35217268	0.68251568
H	-0.24026154	3.42491934	-0.21748883
H	-2.44792472	2.56481640	-0.86134994
H	-2.93227551	0.13791097	-0.74596620
H	-1.18503090	-1.44023984	0.02803912
H	1.03899466	-0.60467240	0.69234335
H	2.13233634	1.62486384	1.76044268
H	3.67993816	0.67183292	0.35216585
H	2.65986910	0.94008421	-1.11166407

2-phenyl-2-propanol, 2P2P (22 atoms)

C	-1.31918548	1.04863817	0.20993456
C	-2.50609195	0.18728046	0.06173141
C	-2.37533701	-1.10869776	-0.42179428
C	-1.04960057	-1.61976394	-0.79461836
C	0.07374332	-0.80650121	-0.67255285
C	-0.03964420	0.58170622	-0.15220482
C	1.22765874	1.43762626	-0.07516273
C	0.95130443	2.89955068	0.34463937
C	2.20325088	0.80708277	0.94129510
O	1.81462570	1.44020763	-1.35591929
H	2.67225804	1.93932851	-1.30539848
H	1.89226304	3.49099104	0.34096426
H	0.52798512	2.94484350	1.37168811
H	0.25157616	3.38295168	-0.36964210
H	1.72798320	0.73351698	1.94318396
H	2.51529970	-0.20910812	0.62080452
H	3.12204234	1.42461218	1.03436831
H	-1.47790474	2.04457760	0.59493642
H	-3.48444571	0.56826946	0.32535637
H	-3.24878318	-1.73850632	-0.53105650
H	-0.95025212	-2.62794806	-1.17508271
H	1.03669543	-1.19720687	-0.97504780

Cumene hydroperoxide, CHP (23 atoms)

C	-0.08719657	3.12576136	-0.05937810
C	-1.24092486	2.40635983	0.26540791
C	-1.16100802	1.04790031	0.56373324
C	0.07507507	0.40533704	0.54569212
C	1.23544771	1.11952364	0.23377953
C	1.18017912	2.49364198	-0.10198856
C	2.47599323	3.29173776	-0.35007001
C	2.19895186	4.58231673	-1.16981904

C	3.49964818	2.43674638	-1.14595746
O	2.99614780	3.61759049	0.92778097
O	4.11162731	4.30856725	0.76156720
H	3.89931862	5.21561368	1.10123595
H	-0.20437750	4.18093210	-0.25268348
H	-2.19893100	2.90931519	0.29952390
H	-2.05531490	0.49621773	0.82319996
H	0.13793363	-0.64739369	0.78971871
H	2.17296174	0.58546187	0.27068754
H	3.01522396	1.96127144	-2.02572738
H	3.95027230	1.65207933	-0.50370882
H	4.34449852	3.05767893	-1.51471532
H	1.66815207	5.33818199	-0.55406668
H	1.59917247	4.34780193	-2.07576643
H	3.14471619	5.05881053	-1.50669770

14. References

1 J. Wm. Wackerly and J. F. Dunne, *J. Chem. Educ.*, **2017**, 94, 1790–1793.

2 W. C. Knol, B. W. J. Pirok and R. A. H. Peters, *Jou. of Sep. Sci.*, **2021**, 44, 63–87.

3 H. Okamura, H. Nishimura, T. Nagata, T. Kigawa, T. Watanabe and M. Katahira, *Sci. Rep.*, **2016**, 6, 21742.

4 J. C. Randall, Ed., *NMR and Macromolecules: Sequence, Dynamic, and Domain Structure*, American Chemical Society, Washington, D.C., **1984**, 247.

5 T. Tseng, Y. Tsai and J. Lee, *Polym. Degrad. and Stab.*, **1997**, 58, 241–245.

6 M. E. Levin, N. O. Gonzales, L. W. Zimmerman and J. Yang, *J. Hazard. Mat.*, 2006, **130**, 88–106.

7 R. Calmanti, N. Sargentoni, M. Selva and A. Perosa, *Catalysts*, 2021, **11**, 1477.

8 H. Phan, P. De La Cruz-Sánchez, M. J. Cabrera-Afonso and B. Martín-Matute, *Green Chem.*, 2025, **27**, 2439–2448.

9 A. Piccinini, S. A. Kavanagh, P. B. Connon and S. J. Connon, *Org. Lett.*, 2010, **12**, 608–611.

10 V. M. Lombardo, E. A. Dhulst, E. K. Leitsch, N. Wilmot, W. H. Heath, A. P. Gies, M. D. Miller, J. M. Torkelson and K. A. Scheidt, *Eur J Org Chem*, 2015, 2791–2795.

11 A. Adronov, https://chemistry.mcmaster.ca/adronov/resources/Stains_for_Developing_TLC_Plates.pdf (accessed on 22-10-2025)

12 K. L. Lin and T. E. Müller, *ACS Sustain. Chem. Eng.*, 2025, **13**, 14792-14803.

13 J. Sun, S.-i. Fujita and B. M. Bhanage, M. Arai, *Cat. Tod.*, **93-95**, 383-388.

14 J. Liu, G. Yang, Y. Liu, D. Wu, X. Hu and Z. Zhang, *Green Chem.*, 2019, **21**, 3834.

15 T. Zhao, G. Long, H. Liang, W. Xiong, X. Hu, *Micropor. Mesopor. Mat.*, 2023, **356**, 112576.
